# Genomics of Ecological Adaptation in Cactophilic *Drosophila*

**DOI:** 10.1093/gbe/evu291

**Published:** 2014-12-31

**Authors:** Yolanda Guillén, Núria Rius, Alejandra Delprat, Anna Williford, Francesc Muyas, Marta Puig, Sònia Casillas, Miquel Ràmia, Raquel Egea, Barbara Negre, Gisela Mir, Jordi Camps, Valentí Moncunill, Francisco J. Ruiz-Ruano, Josefa Cabrero, Leonardo G. de Lima, Guilherme B. Dias, Jeronimo C. Ruiz, Aurélie Kapusta, Jordi Garcia-Mas, Marta Gut, Ivo G. Gut, David Torrents, Juan P. Camacho, Gustavo C.S. Kuhn, Cédric Feschotte, Andrew G. Clark, Esther Betrán, Antonio Barbadilla, Alfredo Ruiz

**Affiliations:** ^1^Departament de Genètica i de Microbiologia, Universitat Autònoma de Barcelona, Spain; ^2^Department of Biology, University of Texas at Arlington; ^3^Institut de Biotecnologia i de Biomedicina, Universitat Autònoma de Barcelona, Spain; ^4^EMBL/CRG Research Unit in Systems Biology, Centre for Genomic Regulation (CRG), Barcelona, Spain; ^5^Universitat Pompeu Fabra (UPF), Barcelona, Spain; ^6^IRTA, Centre for Research in Agricultural Genomics (CRAG) CSIC-IRTA-UAB-UB, Campus UAB, Edifici CRAG, Barcelona, Spain; ^7^The Peter MacCallum Cancer Centre, East Melbourne, Victoria, Australia; ^8^Centro Nacional de Análisis Genómico (CNAG), Parc Científic de Barcelona, Torre I, Barcelona, Spain; ^9^Barcelona Supercomputing Center (BSC), Edifici TG (Torre Girona), Barcelona, Spain and Institució Catalana de Recerca i Estudis Avançats (ICREA), Barcelona, Spain; ^10^Departamento de Genética, Facultad de Ciencias, Universidad de Granada, Spain; ^11^Instituto de Ciências Biológicas, Departamento de Biologia Geral, Universidade Federal de Minas Gerais, Belo Horizonte, MG, Brazil; ^12^Informática de Biossistemas, Centro de Pesquisas René Rachou—Fiocruz Minas, Belo Horizonte, MG, Brazil; ^13^Department of Human Genetics, University of Utah School of Medicine; ^14^Department of Molecular Biology and Genetics, Cornell University

**Keywords:** cactophilic *Drosophila*, genome sequence, ecological adaptation, positive selection, orphan genes, gene duplication

## Abstract

Cactophilic *Drosophila* species provide a valuable model to study gene–environment interactions and ecological adaptation. *Drosophila buzzatii* and *Drosophila mojavensis* are two cactophilic species that belong to the *repleta* group, but have very different geographical distributions and primary host plants. To investigate the genomic basis of ecological adaptation, we sequenced the genome and developmental transcriptome of *D. buzzatii* and compared its gene content with that of *D. mojavensis* and two other noncactophilic *Drosophila* species in the same subgenus. The newly sequenced *D. buzzatii* genome (161.5 Mb) comprises 826 scaffolds (>3 kb) and contains 13,657 annotated protein-coding genes. Using RNA sequencing data of five life-stages we found expression of 15,026 genes, 80% protein-coding genes, and 20% noncoding RNA genes. In total, we detected 1,294 genes putatively under positive selection. Interestingly, among genes under positive selection in the *D. mojavensis* lineage, there is an excess of genes involved in metabolism of heterocyclic compounds that are abundant in *Stenocereus cacti* and toxic to nonresident *Drosophila* species. We found 117 orphan genes in the shared *D. buzzatii–D. mojavensis* lineage. In addition, gene duplication analysis identified lineage-specific expanded families with functional annotations associated with proteolysis, zinc ion binding, chitin binding, sensory perception, ethanol tolerance, immunity, physiology, and reproduction. In summary, we identified genetic signatures of adaptation in the shared *D. buzzatii–D. mojavensis* lineage, and in the two separate *D. buzzatii and D. mojavensis* lineages. Many of the novel lineage-specific genomic features are promising candidates for explaining the adaptation of these species to their distinct ecological niches.

## Introduction

*Drosophila* species are saprophagous insects that feed and breed on a variety of fermenting plant materials, chiefly fruits, flowers, slime fluxes, decaying bark, leaves and stems, cactus necroses, and fungi (Carson 1971). These substrates include bacteria and yeasts that decompose the plant tissues and contribute to the nutrition of larvae and adults (Starmer 1981; Begon 1982). Only two species groups use cacti as their primary breeding site: *repleta *(Oliveira et al. 2012) and *nannoptera* (Lang et al. 2014). Both species groups originated at the *virilis–repleta* radiation, 20–30 Ma (Throckmorton 1975; Morales-Hojas and Vieira 2012; Oliveira et al. 2012) but adapted independently to the cactus niche. The “cactus-yeast-*Drosophila* system” in arid zones provides a valuable model to investigate gene–environment interactions and ecological adaptation from genetic and evolutionary perspectives (Barker and Starmer 1982; Barker et al. 1990). Rotting cacti provide relatively abundant, predictable, and long-lasting resources that can sustain very large *Drosophila* populations. For instance, a single saguaro rot may weigh up to several tons, last for many months, and sustain millions of *Drosophila* larvae and adults (Breitmeyer and Markow 1998). On the other hand, cacti are usually found in arid climates with middle to high temperatures that may impose desiccation and thermal stresses (Loeschcke et al. 1997; Hoffmann et al. 2003; Rajpurohit et al. 2013). Finally, some cacti may contain allelochemicals that can be toxic for *Drosophila* (see below). Thus, adaptation to use cacti as breeding sites must have entailed a fairly large number of changes in reproductive biology, behavior, physiology, and biochemistry (Markow and O’Grady 2008).

**Fig. 1.—:**
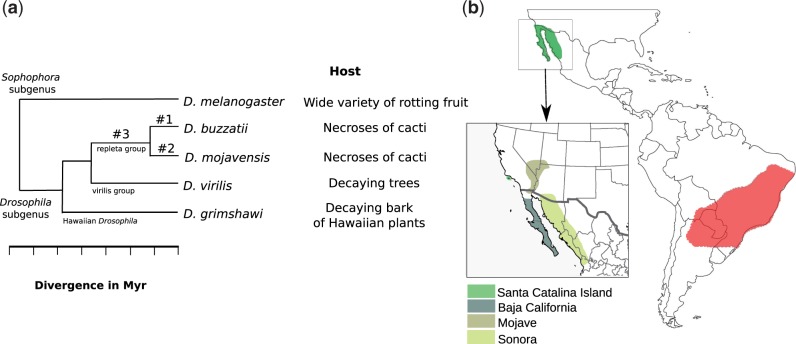
(*a*) Phylogenetic relationship of fruit fly species considered in our comparative analysis and their host preference. (*b*) Geographical distribution of cactophilic species *D. buzzatii* (red) and *D. mojavensis* (green) in America.

We have sequenced the genome and developmental transcriptome of *Drosophila buzzatii* to carry out a comparative analysis with those of *Drosophila mojavensis*, *Drosophila virilis,* and *Drosophila grimshawi* (*Drosophila* 12 Genomes Consortium et al. 2007). *Drosophila buzzatii* and *D. mojavensis* are both cactophilic species that belong to the *mulleri* subgroup of the *repleta* group (Wasserman 1992; Oliveira et al. 2012), although they have very different geographical distributions and host plants (fig. 1). *Drosophila buzzatii* is a subcosmopolitan species which is found in four of the six major biogeographic regions (David and Tsacas 1980). This species is originally from Argentina and Bolivia but now has a wide geographical distribution that includes other regions of South America, the Old World, and Australia (Carson and Wasserman 1965; Fontdevila et al. 1981; Hasson et al. 1995; Manfrin and Sene 2006). It chiefly feeds and breeds in rotting tissues of several Opuntia cacti but can also occasionally use columnar cacti (Hasson et al. 1992; Ruiz et al. 2000; Oliveira et al. 2012). The geographical dispersal of Opuntia by humans in historical times is considered the main driver of the world-wide expansion of *D. buzzatii* (Fontdevila et al. 1981; Hasson et al. 1995).

On the other hand, *D. mojavensis *is endemic to the deserts of Southwestern United States and Northwestern Mexico. Its primary host plants are *Stenocereus gummosus* (pitaya agria) in Baja California and *Stenocereus thurberi *(organ pipe) in Arizona and Sonora*,* but uses also *Ferocactus cylindraceous* (California barrel) in Southern California and *Opuntia *sp. in Santa Catalina Island (Fellows and Heed 1972; Heed and Mangan 1986; Ruiz and Heed 1988; Etges et al. 1999). The ecological conditions of the Sonoran Desert are extreme (dry, arid, and hot), as attested by the fact that only four *Drosophila* species are endemic (Heed and Mangan 1986). In addition, *D. mojavensis* chief host plants, pitaya agria and organ pipe, are chemically complex and contain large quantities of triterpene glycosides, unusual medium-chain fatty acids, and sterol diols (Kircher 1982; Fogleman and Danielson 2001). These allelochemicals are toxic to nonresident *Drosophila* species, decreasing significantly larval performance (Fogleman and Kircher 1986; Ruiz and Heed 1988; Fogleman and Armstrong 1989; Frank and Fogleman 1992). In addition, host plant chemistry and fermentation byproducts affect adult epicuticular hydrocarbons and mating behavior (Havens and Etges 2013) as well as expression of hundreds of genes (Matzkin et al. 2006; Etges et al. 2015; Matzkin 2014).

As a first step to understand the genetic bases of ecological adaptation, here we compare the genomes of the two cactophilic species with those of two noncactophilic species of the *Drosophila* subgenus: *D. virilis* that belongs to the *virilis* species group and *D. grimshawi* that belongs to the picture wing group of Hawaiian *Drosophila* (fig. 1). The lineage leading to the common ancestor of *D. buzzatii* and *D. mojavensis* after diverging from *D. virilis* (#3 in fig. 1) represents the lineage that adapted to the cactus niche (likely Opuntia; Oliveira et al. 2012), whereas the lineages leading to *D. buzzatii* (#1) and *D. mojavensis* (#2) adapted to the specific niche of each species. We carried out a genome-wide scan for 1) genes under positive selection, 2) lineage-specific genes, and 3) gene-duplications in the three lineages (fig. 1). Based on the results of our comparative analyses, we provide a list of candidate genes that might play a meaningful role in the ecological adaptation of these fruit flies.

## Materials and Methods

We sequenced the genome of a highly inbreed *D. buzzatii* strain, st-1 (Betran et al. 1998). DNA was extracted from male and female adults (Piñol et al. 1988; Milligan 1998). Reads were generated with three different sequencing platforms (supplementary fig. S2 and table S12, Supplementary Material online). The assembly of the genome was performed in three stages (supplementary table S13, Supplementary Material online): Preassembly (Margulies et al. 2005), scaffolding (Boetzer et al. 2011), and gapfilling (Nadalin et al. 2012). In each step, a few chimeric scaffolds were identified and split. The final assembly, named Freeze 1, contains 826 scaffolds greater than 3 kb and N50 and N90 index are 30 and 158, respectively. The distribution of read depth in the preassembly showed a Gaussian distribution with a prominent mode centered at approximately 22× (supplementary fig. S3, Supplementary Material online). CG content is approximately 35% overall, approximately 42% in gene regions (including introns) and reaches approximately 52% in exons (supplementary table S14, Supplementary Material online). Unidentified nucleotides (N’s) represent approximately 9% overall, approximately 4% in gene regions, and 0.004% in exons. Sequence quality was assessed by comparing Freeze 1 with five Sanger sequenced bacterial artificial chromosomes (BACs) (Negre et al. 2005; Prada 2010; Calvete et al. 2012) and with Illumina genomic and RNA sequencing (RNA-Seq) reads (supplementary fig. S4, Supplementary Material online). Quality assessments gave an overall error rate of approximately 0.0005 and a PHRED quality score of approximately Q33 (supplementary tables S15 and S16, Supplementary Material online). An overall proportion of segregating sites of approximately 0.1% was estimated (supplementary table S17, Supplementary Material online).

The genome size of two *D. buzzatii* strains, st-1 and j-19, was estimated by Feulgen Image Analysis Densitometry. The genome size of *D. mojavensis* 15081-1352.22 strain (193,826,310 bp) was used as reference (*Drosophila* 12 Genomes Consortium et al. 2007). Testicles from anesthetized males were dissected in saline solution and fixed in acetic-alcohol 3:1. Double preparations of *D. mojavensis *and *D. buzzatii* were made by crushing the fixed testicles in 50% acetic acid. Following Ruiz-Ruano et al. (2011), the samples were stained by Feulgen reaction and images obtained by optical microscopy were analyzed with the pyFIA software (supplementary fig. S5 and table S18, Supplementary Material online).

The 826 scaffolds in Freeze 1 were assigned to chromosomes by aligning their sequences with the *D. mojavensis* genome using MUMmer (Delcher et al. 2003). In addition, the 158 scaffolds in the N90 index were mapped, ordered, and oriented (supplementary fig. S1, Supplementary Material online) using conserved linkage (Schaeffer et al. 2008), in situ hybridization, and additional information (González et al. 2005; Guillén and Ruiz 2012). To estimate the number of rearrangements between *D. buzzatii* and *D. mojavensis*, their chromosomes were compared using GRIMM (Tesler 2002; Delprat A, Guillén Y, Ruiz A, in preparation). Genes in the *Hox* gene complex (*HOM-C*) and five other gene complexes were searched in silico in the *D. buzzatii* genome and manually annotated using available information (Negre et al. 2005), the annotated *D. mojavensis* and *Drosophila melanogaster* genomes, and the RNA-seq data generated for *D. buzzatii* (Negre B, Muyas F, Guillén Y, Ruiz A, in preparation). Transposable elements (TEs) were annotated with RepeatMasker using a comprehensive TE library compiled from FlyBase (St Pierre et al. 2014), Repbase (Jurka et al. 2005), and RepeatModeler. Tandem Repeats Finder version 4.04 (Benson 1999) was used to identify satellite DNAs (satDNAs).

For the RNA-Seq experiments, RNA from frozen samples (embryos, larvae, pupae, adult males, and adult females) was processed using the TruSeq RNA sample preparation kit provided by Illumina. We used a Hi-Seq2000 Illumina Sequencer to generate nonstrand-specific paired-end approximately 100 bp reads from poly(A) + RNA. Between 60 and 89 million reads were generated per sample. A total of approximately 286 million filtered reads were mapped to Freeze 1 with TopHat (Trapnell et al. 2009) representing approximately 180 × coverage of the total genome size (supplementary table S19, Supplementary Material online). Transcripts were assembled with Cufflinks (Trapnell et al. 2010) using Annotation Release 1 as reference (see below).

Protein-coding genes (PCGs) were annotated combining with EVidence Modeler (EVM; Haas et al. 2008) the results of different predictors: Augustus (Stanke and Waack 2003), SNAP (Korf 2004), N-SCAN (Korf et al. 2001), and Exonerate (Slater and Birney 2005). The EVM set contained 12,102 gene models. We noticed that orthologs for a considerable number of *D. mojavensis* PCGs were absent from this data set. Thus, we used the Exonerate predictions to detect another 1,555 PCGs not reported by EVM (Poptsova and Gogarten 2010). Altogether, we predicted a total of 13,657 PCG models in the *D. buzzatii* reference genome (Annotation Release 1). Features of these models are given in supplementary table S20, Supplementary Material online. The RSD (reciprocal smallest distance) algorithm (Wall and Deluca 2007) was used to identify 9,114 1:1 orthologs between *D. mojavensis* and *D. buzzatii. *Orthology relationships among the four species in the *Drosophila* subgenus (fig. 1) were inferred from *D. buzzatii–D. mojavensis* list of orthologs and the OrthoDB catalog (version 6; Kriventseva et al. 2008). To test for positive selection, we compared different codon substitution models using the likelihood ratio test (LRT). We run two pairs if site models (SM) on the orthologs set between *D. buzzatii* and *D. mojavensis*: M7 versus M8 and M1a versus M2a (Yang 2007). Then, we used branch-site models (BSM) to test for positive selection in three lineages (fig. 1): *D. mojavensis *lineage, *D. buzzatii* lineage, and the lineage that led to the two cactophilic species (*D. buzzatii* and *D. mojavensis*). We run Venny software (Oliveros 2007) to create a Venn diagram showing shared selected genes among the different models. We identified genes that are only present in the two cactophilic species, *D. mojavensis* and *D. buzzatii*, by blasting the amino acid sequences from the 9,114 1:1 orthologs between *D. mojavensis* and *D. buzzatii *(excluding misannotated genes) against all the proteins from the remaining 11 *Drosophila* species available in FlyBase protein database, excluding *D. mojavensis *(St Pierre et al. 2014).

For gene duplication analysis (DNA- and RNA-mediated duplications), we used annotated PCGs from the four species of the *Drosophila* subgenus (see supplementary methods, Supplementary Material online). Briefly, we ran all-against-all BLASTp and selected hits with alignment length extending over at least 50% of both proteins and with amino acid identity of at least 50%. Markov Cluster Algorithm (Enright et al. 2002) was used to cluster retained proteins into gene families. The data set was further modified to include additional family members based on sequence coverage and to exclude family members with internal stop codons and matches to TEs. Gene counts for each family from the four species were analyzed with an updated version of CAFE (CAFE 3.1 provided by the authors; Han et al. 2013) to identify lineage-specific expansions. The sets of CAFE-identified expanded families in the *D. buzzatii* and *D. mojavensis* genomes were examined for the presence of lineage-specific duplications. Families that included members with *d*_*s*_ <0.4 were examined manually and lineage-specific duplications were inferred when no hits were found in the syntenic region of the genome with a missing copy. *Drosophila buzzatii*-specific RNA-mediated duplications were identified by examining intron-less and intron-containing gene family members. A duplicate was considered a retrocopy if its sequence spanned all introns of the parental gene. The number of families identified by CAFE as expanded along the internal cactophilic branch was reduced by considering only those families that were also found in expanded category after rerunning the analysis with a less stringent cutoff (35% amino acid identity, 50% coverage). The overlapping set of expanded families was manually examined to verify the absence of *D. buzzatii* and *D. mojavensis* new family members in the *D. virilis* genome. Functional annotation (i.e., Gene Ontology [GO] term) for all expanded families was obtained using the DAVID annotation tool (Huang et al. 2009a, 2009b). For genes without functional annotation in DAVID, annotations of *D. melanogaster* orthologs were used. An extended version of these methods is given as supplementary methods, Supplementary Material online.

## Results

### Features of the *D. buzzatii* Genome

#### Genome Sequencing and Assembly

We sequenced and de novo assembled the genome of *D. buzzatii* line st-1 using shotgun and paired-end reads from 454/Roche, mate-pair and paired-end reads from Illumina, and Sanger BAC-end sequences (∼22× total expected coverage; see Materials and Methods for details). We consider the resulting assembly (Freeze 1) as the reference *D. buzzatii* genome sequence (table 1). This assembly comprises 826 scaffolds greater than 3 kb long with a total size of 161.5 Mb. Scaffold N50 and N90 indexes are 30 and 158, respectively, whereas scaffold N50 and N90 lengths are 1.38 and 0.16 Mb, respectively (table 1). Quality controls (see Materials and Methods) yielded a relatively low error rate of approximately 0.0005 (PHRED quality score *Q* = 33). For comparison, we also assembled the genome of the same line (st-1) using only four lanes of short (100 bp) Illumina paired-end reads (∼76× expected coverage) and the SOAPdenovo software (Luo et al. 2012). This resulted in 10,949 scaffolds greater than 3 kb long with a total size of 144.2 Mb (table 1). All scaffolds are available for download from the *Drosophila buzzatii* Genome Project web page (http://dbuz.uab.cat, last accessed January 7, 2015). This site also displays all the information generated in this project (see below).

**Table 1:** Summary of Assembly Statistics for the Genome of *Drosophila buzzatii*

Assembly	Freeze 1	SOAPdenovo
Number of scaffolds (>3 kb)	826	10,949
Coverage	∼22×	∼76×
Assembly size (bp)	161,490,851	144,184,967
Scaffold N50 index	30	2,035
Scaffold N50 length (bp)	1,380,942	18,900
Scaffold N90 index	158	7,509
Scaffold N90 length (bp)	161,757	5,703
Contig N50 index	1,895	2,820
Contig N50 length (bp)	17,678	3,101

#### Genome Size and Repeat Content

The genome sizes of two *D. buzzatii* strains, st-1 and j-19, were estimated by Feulgen Image Analysis Densitometry on testis cells (Ruiz-Ruano et al. 2011) using *D. mojavensis* as reference. Integrative Optical Density values were 21% (st-1) and 25% (j-19) smaller than those for *D. mojavensis*. Thus, taking 194 Mb (total assembly size) as the genome size of *D. mojavensis* (*Drosophila* 12 Genomes Consortium et al. 2007) we estimated the genome sizes for *D. buzzatii* st-1 and j-19 lines as 153 and 146 Mb, respectively.

To assess the TE content of the *D. buzzatii* genome, we masked the 826 scaffolds of Freeze 1 assembly using a library of TEs compiled from several sources (see Materials and Methods). We detected a total of 56,901 TE copies covering approximately 8.4% of the genome (table 2). The most abundant TEs seem to be Helitrons, LINEs, long terminal repeat (LTR) retrotransposons, and TIR transposons that cover 3.4%, 1.6%, 1.5%, and 1.2% of the genome, respectively (table 2). In addition, we identified tandemly repeated satDNAs with repeat units longer than 50 bp (Melters et al. 2013) (see Materials and Methods). The two most abundant tandem repeat families are the pBuM189 satellite (Kuhn et al. 2008) and the DbuTR198 satellite, a novel family with repeat units 198 bp long (table 3). The remaining tandem repeats had sequence similarity to integral parts of TEs, such as the internal tandem repeats of the transposon Galileo (de Lima LG, Svartman M, Ruiz A, Kuhn GCS, in preparation).

**Table 2:** Transposable Element Content of *Drosophila buzzatii* Genome

Class	Order	Annotated Base Pair	Genome Coverage (%)
I (retrotransposons)	LTR	2,366,439	1.47
	DIRS	55	0.00
	LINE	2,541,645	1.57
II (DNA transposons)	TIR	2,017,167	1.25
	Helitron	5,531,009	3.42
	Maverick	189,267	0.12
	Unknown	973,759	0.60
Total		13,619,341	8.43

Note.—The classification follows Wicker et al. (2007).

**Table 3:** Satellite DNAs Identified in the *Drosophila buzzatii* Genome

Tandem repeat Family	Repeat Length	GC Content (%)	Genome Coverage (%)^a^	Consensus Sequence^b^	Distribution
pBuM189	189	29	0.039	GCAAAAGACTCCGTCAATTA GAAAACAAAAAATGTTATAGTTTTGAGGATTAACC GGCAAAAACCGTATTATTTGTTATAT GATTTCTGTATGGAATACCGTTTTAGAA GCGTCTTTTATCGTATTACTCAGATATATCT TAAGATTTAGCATAATCTAAGAACTTTT TGAAATATTCACATTTGTCCA	*D. buzzatii* cluster species *D. mojavensis*
DbuTR198	198	34	0.027	AAGGTAGAAAGGTAGTTGGTGAGATAAACCAGAAAAA GAGCTAAAAACGGCTAAAAACGGCTAGAAAATAGCCA GAAAGGTAGATTGAACATTAATGGGCAAATGG ATGGATAAATAAGACTGGTCATCATCCAA TGAACAGAATCATGATTAAGAGATAGAAATA TGATTAGAAAGTAGGATAGAAAGGTTAGAAAG	*D. buzzatii*

^a^Genome fraction was calculated assuming a genome size of 163,547,398 bp (version 1 freeze of all contigs).

^b^Consensus sequence generated after clustering TRF results (see Materials and Methods).

#### Chromosomal Rearrangements

The basic karyotype of *D. buzzatii* is similar to that of the *Drosophila* genus ancestor and consists of six chromosome pairs: Four pairs of equal-length acrocentric autosomes, one pair of “dot” autosomes, a long acrocentric X, and a small acrocentric Y (Ruiz and Wasserman 1993). Because no interchromosomal reorganizations between *D. buzzatii* and *D. mojavensis* have previously been found (Ruiz et al. 1990; Ruiz and Wasserman 1993) all 826 scaffolds were assigned to chromosomes by BLASTn against the *D. mojavensis* genome. In addition, the 158 scaffolds in the N90 index were mapped to chromosomes, ordered, and oriented (supplementary fig. S1, Supplementary Material online; Delprat A, Guillén Y, Ruiz A, in preparation) using conserved linkage (Schaeffer et al. 2008) and additional information (González et al. 2005; Guillén and Ruiz 2012). A bioinformatic comparison of *D. buzzatii* and *D. mojavensis* chromosomes confirmed that chromosome 2 differs between these species by ten inversions (*2m, 2n, 2z*^7^, *2c, 2f, 2g, 2h, 2q, 2r*, and *2s*), chromosomes X and 5 differ by one inversion each (*Xe* and *5g*, respectively), and chromosome 4 is homosequential as previously described (Ruiz et al. 1990; Ruiz and Wasserman 1993; Guillén and Ruiz 2012). In contrast, we find that chromosome 3 differs by five inversions instead of the expected two that were previously identified by cytological analyses (Ruiz et al. 1990). These three additional chromosome 3 inversions seem to be specific to the *D. mojavensis* lineage (Delprat A, Guillén Y, Ruiz A, in preparation). One of these inversions, *3f*^2^, is polymorphic in natural populations of *D. mojavensis*, but, conflicting with previous reports (Ruiz et al. 1990; Schaeffer et al. 2008), appears to be homozygous in the sequenced strain. This has been corroborated by the cytological reanalysis of its polytene chromosomes (Delprat et al. 2014).

Many developmental genes are arranged in gene complexes each comprising a small number of functionally related genes. We checked the organization of six of these gene complexes in the *D. buzzatii* genome: *HOM-C*, *Achaete–scute* complex, *Iroquois* complex, *NK* homeobox gene cluster (*NK-C*), *Enhancer of split* complex, and *Bearded* complex (*Brd-C*) (Negre B, Muyas F, Guillén Y, Ruiz A, in preparation). *Hox* genes were arranged in a single complex in the *Drosophila* genus ancestor (Hughes and Kaufman 2002). However, this *HOM-C* suffered two splits (caused by chromosomal inversions) in the lineage leading to the *repleta* species group (Negre et al. 2005). In order to fully characterize *HOM-C* organization in *D. buzzatii*, we manually annotated all *Hox* genes and located them in three scaffolds (2, 5, and 229) of chromosome 2 (Negre B, Muyas F, Guillén Y, Ruiz A, in preparation). The analysis of these scaffolds revealed that only two clusters of *Hox* genes are present. The distal cluster contains *proboscipedia, Deformed, Sex combs reduced, Antennapedia* and *Ultrabithorax*, whereas the proximal cluster contains *labial, abdominal A* and *Abdominal B*. This is precisely the same *HOM-C* organization observed in *D. mojavensis* (Negre and Ruiz 2007). Therefore, there seem to be no additional rearrangements of the *HOM-C* in *D. buzzatii *besides those already described in the genus *Drosophila* (Negre and Ruiz 2007). The other five developmental gene complexes contain 4, 3, 6, 13, and 6 functionally related genes, respectively (Lai et al. 2000; Garcia-Fernàndez 2005; Irimia et al. 2008; Negre and Simpson 2009). All these complexes seem largely conserved in the *D. buzzatii *genome with few exceptions (Negre B, Muyas F, Guillén Y, Ruiz A, in preparation). The gene *slouch* is separated from the rest of the *NK-C* in *D. buzzatii* and also in all other *Drosophila* species outside of the melanogaster species group; in addition, the gene *Bearded*, a member of the *Brd-C*, is seemingly absent from the *D. buzzatii *and *D. mojavensis *genomes, although it is present in *D. virilis* and *D. grimshawi*. On the other hand, genes flanking the complexes are often variable, presumably due to the fixation of chromosomal inversions with breakpoints in the boundaries of the complexes.

#### PCG Content

We used a combination of ab initio and similarity-based algorithms in order to reduce the high false-positive rate associated with de novo gene prediction (Wang et al. 2003; Misawa and Kikuno 2010) as well as to avoid the propagation of false-positive predicted gene models when closely related species are used as references (Poptsova and Gogarten 2010). A total of 13,657 PCGs were annotated in the *D. buzzatii* genome (Annotation Release 1). These PCG models contain a total of 52,250 exons with an average of 3.8 exons per gene. Gene expression analyses provided transcriptional evidence for 88.4% of these gene models (see below). The number of PCGs annotated in *D. buzzatii* is lower than the number annotated in *D. mojavensis* (14,595, Release 1.3), but quite close to the number annotated in *D. melanogaster *(13,955, Release 5.56), one of the best-known eukaryotic genomes (St Pierre et al. 2014). However PCGs in both *D. buzzatii* and *D. mojavensis* genomes tend to be smaller and contain fewer exons than those in the *D. melanogaster* genome (supplementary table S1, Supplementary Material online), which suggests that the annotation in the two cactophilic species might be incomplete. After applying several quality filters, a total of 12,977 high confidence protein-coding sequences (CDS) were selected for further analysis (see Materials and Methods).

#### Developmental Transcriptome

**Fig. 2.—:**
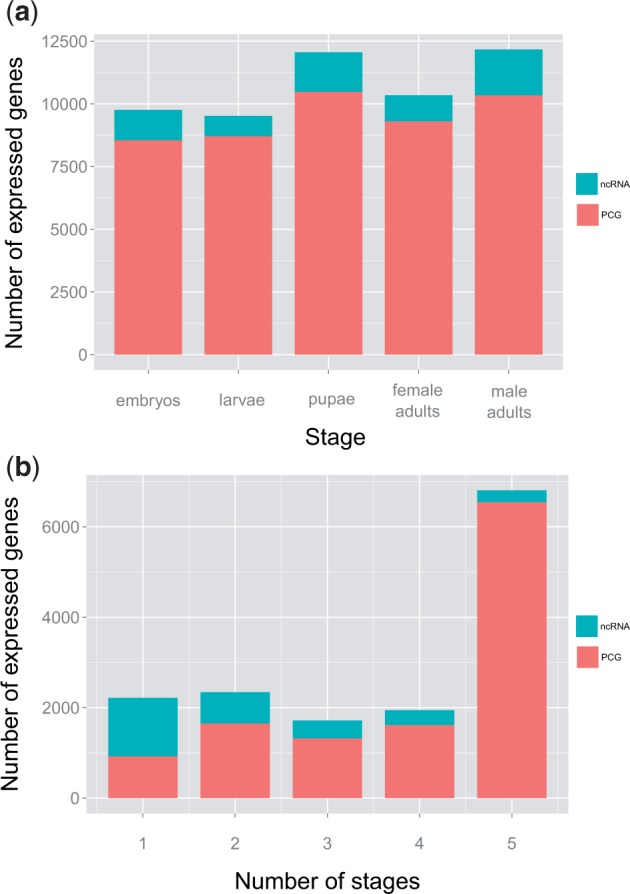
Developmental expression profile of *D. buzzatii *genes*. *(*a*) Number of expressed PCGs (red) and ncRNA genes (blue) along five developmental stages. (*b*) Classification of PCGs and ncRNA genes according to the number of stages where they are expressed.

To characterize the expression profile throughout *D. buzzatii* development, we performed RNA-Seq experiments using samples from five different stages: Embryos, larvae, pupae, adult females, and adult males. Gene expression levels were calculated based on fragments per kilobase of exon per million fragments mapped (FPKM) values. PCG models that did not show evidence of transcription (FPKM < 1) were classified as nonexpressed PCGs, whereas transcribed regions that did not overlap with any annotated PCG model were tentatively considered noncoding RNA (ncRNA) genes (fig. 2*a*). We detected expression (FPKM > 1) of 26,455 transcripts and 15,026 genes, 12,066 (80%) are PCGs and 2,960 (20%) are ncRNA genes. The number of expressed genes (PCGs + ncRNA) increases through the life cycle with a maximum of 12,171 in adult males (fig. 2*a* and supplementary table S2, Supplementary Material online), a pattern similar to that found in *D. melanogaster* (Graveley et al. 2011). In addition, we observed a clear sex-biased expression in adults: Males express 1,824 more genes than females. Previous studies have attributed this sex-biased gene expression mainly to the germ cells, indicating that the differences between ovary and testis are comparable to those between germ and somatic cells (Parisi et al. 2004; Graveley et al. 2011).

We assessed expression breadth for each gene simply as the number of developmental stages with evidence of expression (fig. 2*b* and supplementary table S2, Supplementary Material online). Expression breadth is significantly different (*P* < 0.001) for PCGs and ncRNA genes. A total of 6,546 expressed PCGs (54.2%) are constitutively expressed (i.e., we observed expression in the five stages), but only 260 of ncRNA genes (8.8%) are constitutively expressed (supplementary table S2, Supplementary Material online). In contrast, 925 expressed PCGs (7.7%) and 1,292 ncRNA genes (43.6%) are expressed only in one stage. Mean expression breadth was 3.9 for PCGs and 2.2 for ncRNA genes. Adult males show more stage-specific genes (844 genes) compared with adult females (137 genes).

PCGs with no expression in this study (FPKM < 1) might be expressed at a higher level in other tissues or times, or they might be inducible under specific conditions that we did not test (Weake and Workman 2010; Etges et al. 2015; Matzkin 2014). We also must expect that some remaining fraction of gene models will be false positives (Wang et al. 2003). However, because we used a combination of different annotation methods to reduce the proportion of false-positives, we expect this proportion to be very small. On the other hand, transcribed regions that do not overlap with any annotated PCG models are likely ncRNA genes although we cannot discard that some of them might be false negatives, that is, genes that went undetected by our annotation methods perhaps because they contain small open reading frames (Ladoukakis et al. 2011). One observation supporting that most of them are in fact ncRNA genes is that their expression breadth is quite different from that of PCGs and a high fraction of them are stage-specific genes. In most *Drosophila* species, with limited analyses of the transcriptome (Celniker et al. 2009), few ncRNA genes have been annotated. In contrast, in *D. melanogaster* with a very well-annotated genome, 2,096 ncRNA genes have been found (Release 5.56, FlyBase). Thus, the number of ncRNA found in *D. buzzatii *is comparable to that of *D. melanogaster*.

#### Website

A website (http://dbuz.uab.cat, last accessed January 7, 2015) has been created to provide free access to all information and resources generated in this work. It includes a customized browser (GBrowse; Stein et al. 2002) for the *D. buzzatii *genome incorporating multiple tracks for gene annotations with different gene predictors, for expression levels and transcript annotations for each developmental stage, and for repeat annotations. It contains also utilities to download contigs, scaffolds, and data files and to carry out Blast searches against all *D. buzzatii* contigs and scaffolds.

### Lineage-Specific Analyses

We set up to analyze three lineages for several aspects that could reveal genes involved in adaptation to the cactophilic niche. These lineages are denoted as #1, #2, and #3, respectively, in figure 1: *D. buzzatii* lineage, *D. mojavensis* lineage, and cactophilic lineage (i.e., lineage shared by *D. buzzatii* and *D. mojavensis*). We searched for genes under positive selection, duplicated genes, and orphan genes in those lineages.

#### Genes under Positive Selection

We first searched for genes evolving under positive selection during the divergence between *D. buzzatii* and *D. mojavensis,* using codon substitution models implemented in the PAML 4 package (Yang 2007). Two pairs of different SM were compared by the LRT, M1a versus M2a and M7 versus M8 (see Materials and Methods). In each case, a model that allows for sites with ω > 1 (positive selection) is compared with a null model that considers only sites with ω < 1 (purifying selection) and ω = 1 (neutrality). At *P* < 0.001, the first comparison (M1a vs. M2a) detected 915 genes whereas the second comparison (M7 vs. M8) detected 802 genes. Comparison of the two gene sets allowed us to detect 772 genes present in both, and this was taken as the final list of genes putatively under positive selection using SM (supplementary table S3, Supplementary Material online).

**Fig. 3.—:**
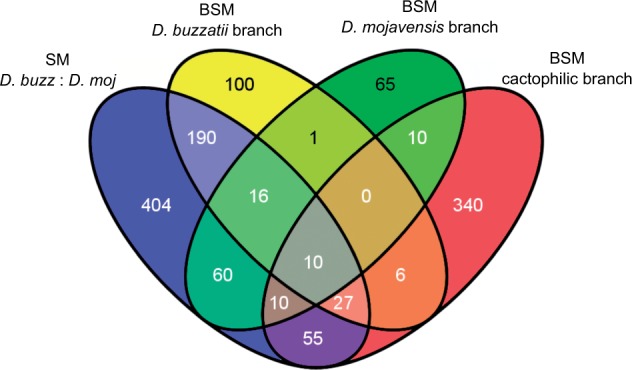
Venn diagram showing the number of genes putatively under positive selection detected by two different methods, SM and BSM using three different lineages as foreground branches.

Next, we used BSM from PAML 4 package (Yang 2007) to search for genes under positive selection in the phylogeny of the four *Drosophila* subgenus species, *D. buzzatii*, *D. mojavensis*, *D. virilis,* and *D. grimshawi* (fig. 1). Orthologous relationships among the four species were inferred from *D. buzzatii–D. mojavensis* list of orthologs and the OrthoDB catalog (see Materials and Methods). A total of 8,328 unequivocal 1:1:1:1 orthologs were included in the comparison of a BSM allowing sites with ω > 1 (positive selection) and a null model that does not. We selected three branches to test for positive selection (the foreground branches): *D. buzzatii* lineage, *D. mojavensis* lineage, and cactophilic lineage (denoted as #1, #2, and #3 in fig. 1). The number of genes putatively under positive selection detected at *P* < 0.001 in the three branches was 350, 172, and 458, respectively (supplementary table S3, Supplementary Material online). These genes only partially overlap those previously detected in the *D. buzzatii*–*D. mojavensis* comparison using SM (fig. 3). Although 69.4% and 55.8% of the genes putatively under positive selection in the *D. buzzatii* and *D. mojavensis *lineages were also detected in the *D. buzzatii*–*D. mojavensis* comparison, only 22.3% of the genes detected in the cactophilic lineage were present in the previous list (fig. 3). Thus, the total number of genes putatively under positive selection is 1,294.

We looked for functional categories overrepresented among the candidate genes reported by both SM and BSM (table 4). We first performed a GO enrichment analysis with the 772 candidate genes uncovered by SM comparing *D. mojavensis* and *D. buzzatii* orthologs using DAVID tools (Huang et al. 2007). Two molecular functions show higher proportion than expected by chance (relative to *D. mojavensis* genome) within the list of candidate genes: Antiporter activity and transcription factor activity. With respect to the biological process, regulation of transcription is the only overrepresented category. A significant enrichment in Src Homology-3 domain was observed. This domain is commonly found within proteins with enzymatic activity and it is associated with protein binding function.

**Table 4:** GO Analysis of Putative Genes under Positive Selection Detected by Both SM and BSM

Codon substitution Models	Lineage (Branch Number)	Number of Candidates	GO enrichment					
			Molecular Function		Biological Process		Interpro Domain	
			ID	Fold Enrichment	ID	Fold Enrichment	ID	Fold Enrichment
SM	*Drosophila buzzatii* versus *Drosophila mojavensis*	772	Antiporter activity	1.77	Regulation of transcription	4.90	Src homology-3 domain	1.60
			Transcription factor activity	1.56				
BSM	*D. buzzatii* #1	350	DNA binding	1.36	Regulation of transcription DNA dependent	1.36	Immunoglobulin-like	1.33
					Phosphate metabolic process	0.72		
	*D. mojavensis* #2	172	Dopamine beta-monooxigenase activity	2.35	Heterocycle catabolic process	2.35	DOMON (DOpamine beta-MOnooxygenase N-terminal domain)	2.35
					Cation transport	0.98		
					Histidine family amino acid catabolic process	2.35		
	Cactophilic #3	458	Zinc ion binding	2.01	Cytoeskeleton organization	1.67	Zinc finger, PHD-type	1.93
			Transition metal ion binding	2.01	Regulation of transcription DNA dependent	1.06	Proteinase inhibitor I1 kazal	2.20
			DNA binding	1.66				

Note.—Only categories showing an enrichment with a *P* value less than 1.0e-03 are included.

A similar GO enrichment analysis was carried out with candidate genes found using BSM in each of the three targeted branches. The 350 candidate genes in *D. buzzatii* lineage show a significant enrichment in DNA-binding function. DNA-dependent regulation of transcription and phosphate metabolic processes were also overrepresented. We also found a significant enrichment in the Ig-like domain, involved in functions related to cell–cell recognition and immune system. The 172 candidate genes in *D. mojavensis* lineage show a significant excess of genes related to the heterocycle catabolic process (*P* = 5.9e-04). Interestingly, the main hosts of *D. mojavensis *(columnar cacti) contain large quantities of triterpene glycosides, which are heterocyclic compounds. Among the candidate genes in the branch leading to the two cactophilic species, there are three overrepresented molecular functions related to both metal and DNA binding. The GO terms with the highest significance in the biological process category are cytoskeleton organization and, once again, regulation of transcription.

Using the RNA-Seq data we determined the expression profiles of all 1,294 genes putatively under positive selection. A total of 1,213 (93.7%) of these genes are expressed in at least one developmental stage (supplementary table S2, Supplementary Material online). A comparison of expression level and breadth between candidate and noncandidate genes revealed that genes putatively under positive selection are expressed at a lower level (Χ^2 ^= 84.96, *P* < 2e-16) and in fewer developmental stages (Χ^2 ^= 26.99, *P* < 2e-6) than the rest.

#### Orphan Genes in the Cactophilic Lineage

**Fig. 4.—:**
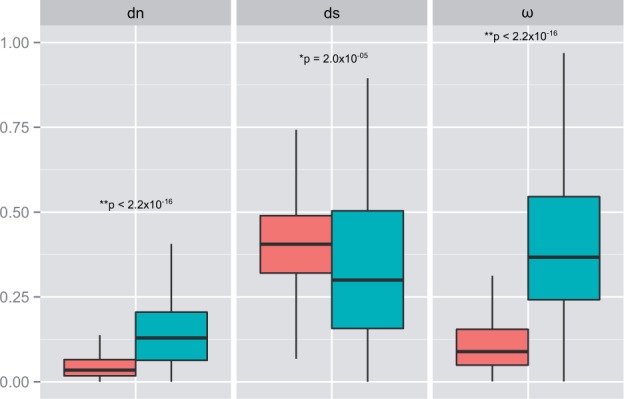
Patterns of divergence in orphan and nonorphan genes. Orphan genes (blue) have significantly higher d*n* and ω values compared with that of nonorphan genes (red). Nonorphan genes show significantly higher d*s*.

To detect orphan genes in the cactophilic lineage, we blasted the amino acid sequences encoded by 9,114 *D. buzzatii* genes with *D. mojavensis* 1:1 orthologs against all proteins from the 12 *Drosophila* genomes except *D. mojavensis* available in FlyBase (St Pierre et al. 2014). We found 117 proteins with no similarity to any predicted *Drosophila* protein (cutoff value of 1e-05) and were considered to be encoded by putative orphan genes. We focused on the evolutionary dynamics of these orphan genes by studying their properties in comparison to the remaining 8,997 1:1 orthologs (fig. 4). We observed that median d*n* of orphan genes was significantly higher than that of nonorphan genes (d*n*_orphan_ = 0.1291; d*n*_non__orphan_ = 0.0341; *W* = 846,254, *P* < 2.2e-16) and the same pattern was observed for ω (ω_orphan_ = 0.4253, ω_non__orphan_ = 0.0887, *W* = 951,117, *P* < 2.2e-16). However, median d*s* of orphan genes is somewhat lower than that for the rest of genes (d*s*_orphan_ = 0.3000, d*s*_non__orphan_ = 0.4056, *W* = 406,799, *P* = 2.4e-05).

We found 19 of the 117 orphan genes in the list of candidate genes detected in the *D. buzzatii–D. mojavensis* comparison (see above). This proportion (16.3%) was significantly higher than that found in nonorphan 1:1 orthologs (753/8,997 = 8.4%), which indicates an association between gene lineage-specificity and positive selection (Fisher exact test, two-tailed, *P* < 0.0001). The 19 orphan genes included in the candidate gene group are not associated with any GO category. As a matter of fact, information about protein domains was found for only two of these genes (GYR and YLP motifs in both cases: GI20994 and GI20995). These results should be viewed cautiously as newer genes are functionally undercharacterized and GO databases are biased against them (Zhang et al. 2012). We also compared the protein length between orphan and nonorphan gene products. Our results showed that orphan genes are shorter (*W* = 68,825.5, *P* < 2.2e-16) and have fewer exons than nonlineage-specific genes (*W* = 201,068, *P* < 2.2e-16).

RNA-Seq data allowed us to test for expression of orphan genes. From the 117 gene candidates, 82 (70%) are expressed at least in one of the five analyzed developmental stages. A comparison of the expression profiles between orphan and the rest of 1:1 orthologous genes showed that the expression breadth of orphans is different from that of nonorphans (Χ^2 ^= 101.4, *P* < 0.001): Most orphan genes are expressed exclusively in one developmental stage with mean expression breadth of 2.56 (vs. 3.94 for nonorphans).

#### Gene Duplications

The annotated PCGs from four species of the *Drosophila* subgenus were used to study gene family expansions in the *D. buzzatii*, *D. mojavensis*, and cactophilic lineages (fig. 1). Proteins that share 50% identity over 50% of their length were clustered into gene families using Markov Cluster Algorithm. After additional quality filters (see Materials and Methods), the final data set consisted of a total of 56,587 proteins from four species clustered into 19,567 families, including single-gene families (supplementary tables S4–S7, Supplementary Material online).

Considering the *D. buzzatii* genome alone (supplementary table S4, Supplementary Material online), we find 11,251 single-copy genes and 1,851 duplicate genes (14%) clustered in 691 gene families. Among *D. buzzatii* gene families, about 70% of families have two members and the largest family includes 16 members (supplementary table S4, Supplementary Material online). Among single-copy genes, 1,786 genes are only present in the *D. buzzatii* lineage. This number decreases only to 1,624 when proteins are clustered into families with a less stringent cutoff of 35% identity and 50% coverage. Such lineage-specific single-copy genes have been found in all the 12 *Drosophila* genomes that have been analyzed, including *D. mojavensis* (Hahn et al. 2007), and although traditionally they have been viewed as annotation artifacts, many of these genes may be either de novo or fast-evolving genes (Reinhardt et al. 2013; Palmieri et al. 2014).

Lineage-specific expansions were identified by analyzing the gene count for each family from the four species using CAFE3.1 (see Materials and Methods). This analysis detected expansions of 86 families along the *D. buzzatii* lineage. However, 15 families increased in size as the result of extra copies added to the data set after taking into account high sequence coverage. The expansions of these families cannot be confirmed with the current genome assembly. The remaining families were analyzed further in order to confirm *D. buzzatii*-specific duplications. To do that, we first selected gene families with members that have d*s* < 0.4 (median d*s* for *D. mojavensis*–*D. buzzatii* orthologs) and then manually examined syntenic regions in *D. mojavensis* genome. Although this approach might miss some true lineage-specific expansions, it reduces the possibility of including old families into the expansion category that might have been misclassified as a result of incomplete gene annotation in the genomes under study or independent loss of family members in different lineages. Of the 30 gene families whose members had d*s* < 0.4, we confirmed the expansion of 20 families (supplementary table S8, Supplementary Material online). In 12 of the 20 families, new family members are found on the same scaffold in close proximity suggesting unequal crossing over or proximate segmental duplication as the mechanisms for duplicate formation. The remaining eight families contain dispersed duplicates found in different scaffolds. Six of these families expanded through retroposition, the RNA-mediated duplication mechanism that allows insertion of reverse-transcribed mRNA nearly anywhere in the genome. In most cases, family expansions are due to addition of a new single copy in the *D. buzzatii* lineage (in 25 of total 35 families). Two families that expanded the most, with up to 5 (Family 95) and 9 (Family 126) new members, encode various peptidases involved in protein degradation. Other expanded families are associated with a broad range of functions, including structural proteins of insect cuticle and chorion, enzymes involved in carbohydrate and lipid metabolism, proteins that function in immune response, and olfactory receptors. In addition, Family 128 encodes female reproductive peptidases (Kelleher and Markow 2009) and it appears that new family members have been acquired independently in *D. buzzatii* and *D. mojavensis* lineages (supplementary table S11, Supplementary Material online).

We find six families in *D. buzzatii* that expanded through retroposition in the 11 Myr since the split between *D. buzzatii* and *D. mojavensis* (supplementary table S9, Supplementary Material online). This gives a rate of 0.55 retrogenes/Myr, which is consistent with previous estimates of functional retrogene formation in *Drosophila* of 0.5 retrogenes/Myr (Bai et al. 2007). The expression of all but one retrogene is supported by RNA-Seq data, with no strong biases in expression between the sexes. Four retrogenes are duplicates of ribosomal proteins, and the parental genes from two of these families (*RpL37a* and *RpL30*) have been previously shown to generate retrogenes in other *Drosophila* lineages (Bai et al. 2007; Han and Hahn 2012). Frequent retroposition of ribosomal proteins could be explained by the high levels of transcription of ribosomal genes although other *Drosophila* lineages do not show a bias in favor of retroduplication of ribosomal proteins (Bai et al. 2007; Han and Hahn 2012). The remaining two retrogenes include the duplicate of Caf1, protein that is involved in histone modification, and the duplicate of VhaM9.7-b, a subunit of ATPase complex.

CAFE analysis identified 127 families that expanded along the *D. mojavensis* lineage. Of these families, 86 contain members with d*s* < 0.4. Further examination of syntenic regions confirmed expansion of only 17 families (supplementary table S8, Supplementary Material online). New members in two families (Families 1121 and 1330) are found in different scaffolds and originated through RNA-mediated duplications. These instances have been previously identified as *D. mojavensis*-specific retropositions (Han and Hahn 2012). Members of expanded families encode proteins that function in proteolysis, peptide and ion transport, aldehyde and carbohydrate metabolism, as well as sensory perception (supplementary table S11, Supplementary Material online). At least 4 of the 17 expanded families play a role in reproductive biology: Proteases of Family 128 with three new members have been shown to encode female reproductive peptidases (Kelleher and Markow 2009), and members of three additional families (Families 187, 277, and 1234) encode proteins that are found in *D. mojavensis* accessory gland proteome (Kelleher et al. 2009).

There are 20 gene families that expanded along the cactophilic branch, that is, before the split between *D. buzzatii *and *D. mojavensis* (see Materials and Methods; supplementary table S10, Supplementary Material online). Most families (16 of 20) have expanded through tandem or nearby segmental duplication and are still found within the same scaffold. The remaining families with dispersed duplicates included one retrogene, the duplicate of T-cp1, identified previously in *D. mojavensis* lineage (Han and Hahn 2012). The extent of per-family expansions in the cactophilic lineage is modest, with two new additional members found in four families and a single new copy in the remaining families. Members of the most expanded families encode guanylate cyclases that are involved in intracellular signal transduction, peptidases, and carbon–nitrogen hydrolases. Members of other families include various proteins with metal-binding properties as well as proteins with a role in vesicle and transmembrane transport (supplementary table S11, Supplementary Material online). We also see expansion of three families (Family 775, Family 776, and Family 800) with functions related to regulation of juvenile hormone (JH) levels (see Discussion).

## Discussion

### The *D. buzzatii* Genome

*Drosophila* is a leading model for comparative genomics, with 24 genomes of different species already sequenced (Adams et al. 2000; *Drosophila* 12 Genomes Consortium et al. 2007; Zhou et al. 2012; Zhou and Bachtrog 2012; Fonseca et al. 2013; Ometto et al. 2013; Chen et al. 2014). However, only five of these species belong to the species-rich *Drosophila* subgenus, and only one of these species, *D. mojavensis*, is a cactophilic species from the large *repleta* species group. Here we sequenced the genome and transcriptome of *D. buzzatii*, another cactophilic member of the *repleta* group, to investigate the genomic basis of adaptation to this distinct ecological niche. Using different sequencing platforms and a three-stage de novo assembly strategy, we generated a high quality genome sequence that consists of 826 scaffolds greater than 3 kb (Freeze 1). A large portion (>90%) of the genome is represented by 158 scaffolds with a minimum size of 160 kb that have been assigned, ordered, and oriented in the six chromosomes of the *D. buzzatii* karyotype. As expected, the assembly is best for chromosome 2 (because of the use of Sanger generated BAC-end sequences) and worst for chromosome X (because of the three-fourth representation of this chromosome in adults of both sexes). The quality of our Freeze 1 assembly compares favorably with the assembly generated using only Illumina reads and the SOAPdenovo assembler, and with those of other *Drosophila* genomes generated using second-generation sequencing platforms (Zhou et al. 2012; Zhou and Bachtrog 2012; Fonseca et al. 2013; Ometto et al. 2013; Chen et al. 2014), although our Freeze 1 does not attain the quality of the 12 *Drosophila* genomes generated using Sanger only (*Drosophila* 12 Genomes Consortium et al. 2007).

*Drosophila buzzatii* is a subcosmopolitan species that has been able to colonize four of the six major biogeographical regions (David and Tsacas 1980). Only two other *repleta* group species (*Drosophila repleta* and *Drosophila hydei*) have reached such widespread distribution. Invasive species are likely to share special genetic traits that enhance their colonizing ability (Parsons 1983; Lee 2002). From an ecological point of view we would expect colonizing species to be r-strategists with a short developmental time (Lewontin 1965). Because there is a correlation between developmental time and genome size (Gregory and Johnston 2008), colonizing species are also expected to have a small genome size (Lavergne et al. 2010). The genome size of *D. buzzatii* was estimated in our assembly as 161 Mb and by cytological techniques as 153 Mb, approximately 20% smaller than the *D. mojavensis* genome. The genome size of a second *D. buzzatii* strain, estimated by cytological techniques, is even smaller, 146 Mb. However, the relationship between genome size and colonizing ability does not hold in the *Drosophila* genus at large. Although colonizing species such as *D. melanogaster *and *Drosophila simulans* have relatively small genomes, specialist species with a narrow distribution such as *Drosophila sechelia* and *Drosophila erecta* also have small genomes. On the other hand, *Drosophila ananassae, Drosophila malerkotliana, Drosophila suzuki, D. virilis*, and *Zaprionus indianus* are also colonizing *Drosophila* species but have relatively large genomes (Nardon et al. 2005; Bosco et al. 2007; *Drosophila* 12 Genomes Consortium et al. 2007; Gregory and Johnston 2008). Further, there seems to be little difference in genome size between original and colonized populations within species (Nardon et al. 2005). Seemingly, other factors such as historical or chance events, niche dispersion, genetic variability, or behavioral shifts are more significant than genome size in determining the current distribution of colonizing species (Markow and O’Grady 2008).

TE content in the *D. buzzatii* genome was estimated as 8.4% (table 2), a relatively low value compared with that of *D. mojavensis*, 10–14% (Ometto et al. 2013; Rius et al., in preparation). These data agree well with the smaller genome size of *D. buzzatii* because genome size is positively correlated with the contribution of TEs (Kidwell 2002; Feschotte and Pritham 2007). However, TE copy number and coverage estimated in *D. buzzatii* (table 2) must be taken cautiously. Coverage is surely underestimated due to the difficulties in assembling repeats, in particular with short sequence reads, whereas the number of copies may be overestimated due to copy fragmentation (Rius N, Guillén Y, Kapusta A, Feschotte C, Ruiz A, in preparation). The contribution of satDNAs (table 3) is also an underestimate and further experiments are required for a correct assessment of this component (de Lima LG, Svartman M, Ruiz A, Kuhn GCS, in preparation). However, we identified the pBuM189 satDNA as the most abundant tandem repeat of *D. buzzatii*. Previous in situ hybridization experiments revealed that pBuM189 copies are located in the centromeric region of all chromosomes, except chromosome X (Kuhn et al. 2008). Thus, pBuM189 satellite is likely the main component of the *D. buzzatii* centromere. Interestingly, a pBuM189 homologous sequence has recently been identified as the most abundant tandem repeat of *D. mojavensis *(Melters et al. 2013). Although the chromosome location in *D. mojavensis* has not been determined, the persistence of pBuM189 as the major satDNA in *D. buzzatii* and *D. mojavensis* may reflect a possible role for these sequences in centromere function (Ugarković 2009).

### Chromosome Evolution

The chromosomal evolution of *D. buzzatii *and *D. mojavensis* has been previously studied by comparing the banding pattern of the salivary gland chromosomes (Ruiz et al. 1990; Ruiz and Wasserman 1993). *Drosophila buzzatii* has few fixed inversions (*2m, 2n, 2z*^7^, and *5g*) when compared with the ancestor of the *repleta* group. In contrast, *D. mojavensis* showed ten fixed inversions (*Xe, 2c, 2f, 2g, 2h, 2q, 2r, 2s, 3a,* and *3d*), five of them (*Xe, 2q, 2r, 2s,* and *3d*) exclusive to *D. mojavensis* and the rest shared with other cactophilic *Drosophila* (Guillén and Ruiz 2012). Thus, the *D. mojavensis* lineage appears to be a derived lineage with a relatively high rate of rearrangement fixation. Here, we compared the organization of both genomes corroborating all known inversions in chromosomes X, 2, 4, and 5. In *D. mojavensis* chromosome 3, however, we found five inversions instead of the two expected (Delprat A, Guillén Y, Ruiz A, in preparation). One of the three additional inversions is the polymorphic inversion *3f*^2^ (Ruiz et al. 1990). This inversion has previously been found segregating in Baja California and Sonora (Mexico) and is homozygous in the strain of Santa Catalina Island (California) that was used to generate the *D. mojavensis* genome sequence (*Drosophila* 12 Genomes Consortium et al. 2007). Previously, the Santa Catalina Island population was thought to have the standard (ancestral) arrangements in all chromosomes, like the populations in Southern California and Arizona (Ruiz et al. 1990; Etges et al. 1999). The presence of inversion *3f*^2^ in Santa Catalina Island is remarkable because it indicates that the flies that colonized this island came from Baja California and are derived instead of ancestral with regard to the rest of *D. mojavensis* populations (Delprat et al. 2014). The other two additional chromosome 3 inversions are fixed in the *D. mojavensis* lineage and emphasize its rapid chromosomal evolution. Guillén and Ruiz (2012) analyzed the breakpoint of all chromosome 2 inversions fixed in *D. mojavensis *and concluded that the numerous gene alterations at the breakpoints with putative adaptive consequences point directly to natural selection as the cause of *D. mojavensis *rapid chromosomal evolution. The four fixed chromosome 3 inversions provide an opportunity for further testing this hypothesis (Delprat A, Guillén Y, Ruiz A, in preparation).

### Candidate Genes under Positive Selection and Orphan Genes

Several methods have been developed to carry out genome-wide scans for genes evolving under positive selection (Nielsen 2005; Anisimova and Liberles 2007; Vitti et al. 2013). We used here a rather simple approach based on the comparison of the nonsynonymous substitution rate (d*n*) with the synonymous substitution rate (d*s*) at the codon level (Yang et al. 2000; Wong et al. 2004; Zhang et al. 2005; Yang 2007). Genes putatively under positive selection were detected on the basis of statistical evidence for a subset of codons where replacement mutations were fixed faster than mutation at silent sites. Four species of the *Drosophila* subgenus (fig. 1) were employed to search for genes under positive selection using SM and BSM. We restricted the analysis to this subset of the *Drosophila* phylogeny to avoid the saturation of synonymous substitutions expected with phylogenetically very distant species (Bergman et al. 2002; Larracuente et al. 2008), and also because these are the genomes with the highest quality available (Schneider et al. 2009). A total of 1,294 candidate genes were detected with both SM and BSM, which represents approximately 14% of the total set of 1:1 orthologs between *D. mojavensis* and *D. buzzatii*. Positive selection seems pervasive in *Drosophila* (Sawyer et al. 2007; Singh et al. 2009; Sella et al. 2009; Mackay et al. 2012) and, using methods similar to ours, it has been estimated that 33% of single-copy orthologs in the *melanogaster* group have experienced positive selection (*Drosophila* 12 Genomes Consortium et al. 2007). The smaller fraction of genes putatively under positive selection in our analyses may be due to the fewer lineages considered in our study. In addition, both studies may be underestimating the true proportion of positively selected genes because only 1:1 orthologs were included in the analyses and genes that evolve too fast may be missed by the methods used to establish orthology relationships (Bierne and Eyre-Walker 2004). At any rate, the 1,294 candidate genes found here should be evaluated using other genomic methods for detecting positive selection, for example, those comparing levels of divergence and polymorphism (Vitti et al. 2013). Furthermore, functional follow-up tests will be necessary for a full validation of their adaptive significance (Lang et al. 2012).

BSM allowed us to search for positively selected genes in the three-targeted lineages (*D. buzzatii*, *D. mojavensis,* and cactophilic branch). We then performed GO enrichment analyses in order to identify potential candidates for environmental adaptation given the ecological properties of both cactophilic species (table 4). The most interesting result of this analysis is that genes putatively under positive selection in *D. mojavensis* branch are enriched in genes involved in heterocyclic catabolic processes. Four candidate *D. mojavensis* genes, *GI19101, GI20678, GI21543* and *GI22389,* that are orthologous to *D. melanogaster* genes *nahoda*, *CG5235*, *slgA* and *knk*, respectively, participate in these processes and might be involved in adaptation of *D. mojavensis* to the *Stenocereus cacti*, plants with particularly large quantities of heterocyclic compounds (see Introduction). A difficulty with this interpretation is the fact that the *D. mojavensis* genome sequence was generated using a strain from Santa Catalina Island where *D. mojavensis* inhabits Opuntia cactus (*Drosophila* 12 Genomes Consortium et al. 2007). However, the evidence indicates that the ancestral *D. mojavensis* population is the agria-inhabiting Baja California population and that the Mainland Sonora population split from Baja California approximately 0.25 Ma whereas the Mojave Desert and Mainland Sonora populations diverged more recently, approximately 0.125 Ma (Smith et al. 2012). Moreover, the presence of inversion *3f*^2^ in the Santa Catalina Island population suggests that the flies that colonized this island came from Baja California populations, where this inversion is currently segregating, and not from the Mojave Desert, where this inversion is not present (Delprat et al. 2014). This is compatible with mitochondrial DNA sequence data (Reed et al. 2007) although in contrast to other data (Machado et al. 2007). Finally, the transcriptional profiles of the four *D. mojavensis* subpopulations reveal only minor gene expression differences between individuals from Santa Catalina Island and Baja California (Matzkin and Markow 2013).

Orphan genes are genes with restricted taxonomic distribution. Such genes have been suggested to play an important role in phenotypic and adaptive evolution in multiple species (Domazet-Lošo and Tautz 2003; Khalturin et al. 2009; Chen et al. 2013). The detection of orphan genes is highly dependent on the availability of sequenced and well-annotated genomes of closely related species, and the total number of lineage-specific genes tend to be overestimated (Khalturin et al. 2009). We were as conservative as possible by considering only high-confidence 1:1 orthologs in two species, *D. buzzatii* and *D. mojavensis*. The result is a set of 117 orphans in the cactophilic lineage.

We observe that orphan genes clearly show a different pattern of molecular evolution compared with that of older conserved genes. Orphans exhibit a higher d*n* that can be attributed to more beneficial mutations fixed by positive selection or to lower constraint, or both (Cai and Petrov 2010; Chen et al. 2010). However, as the number of genes putatively under positive selection within the set of orphan genes is higher than expected by chance, we suggest that the elevated d*n* likely reflects adaptive evolution.

Orphans also have fewer exons and encode shorter proteins than nonorphans. This observation has been reported in multiple eukaryotic organisms such as yeasts (Carvunis et al. 2012), fruitflies (Domazet-Lošo and Tautz 2003) and primates (Cai and Petrov 2010), and it is further supported by a positive correlation between protein length and sequence conservation (Lipman et al. 2002) (see above). We did not find expression support for all the orphan genes detected. This suggests to us that either orphans are more tissue- or stage-specific than nonorphans (Zhang et al. 2012) or we are actually detecting artifactual CDS that are not expressed. However, given the patterns of sequence evolution of orphan genes, we favor the first explanation for the majority of them. Collectively, all these results support the conclusion that orphan genes evolve faster than older genes, and that they experience lower levels of purifying selection and higher rates of adaptive evolution (Chen et al. 2010).

It has been widely reported that younger genes have lower expression levels than older genes on average (Cai and Petrov 2010; Tautz and Domazet-Lošo 2011; Zhang et al. 2012). Here, we observe that orphan genes that are being transcribed are less expressed than nonorphans (Kruskal test, Χ^2 ^= 9.37, *P* = 0.002). One of the proposed hypotheses to explain these observations is that genes that are more conserved are indeed involved in more functions (Pál et al. 2006; Tautz and Domazet-Lošo 2011).

Different studies have demonstrated that newer genes are more likely to have stage-specific expression than older genes (Zhang et al. 2012). Here, we show that the number of stage-specific expressed orphans is significantly higher than that of older genes. It has been proposed that newer genes tend to be more developmentally regulated than older genes (Tautz and Domazet-Lošo 2011). This means that they contribute most to the ontogenic differentiation between taxa (Chen et al. 2010). In *D. buzzatii* the vast majority of stage-specific orphan genes are expressed in larvae (15/29), indicating that expression of younger genes is mostly related to stages in which *D. buzzatii* and *D. mojavensis* lineages most diverge from each other.

### Gene Duplication

The study of gene duplications in the *D. buzzatii* and *D. mojavensis *lineages aims at understanding the genetic bases of the ecological specialization associated with colonization of novel cactus habitats. Although we only considered expanded families, it is known that specialization sometimes involves gene losses. For example, *D. sechellia* and *D. erecta, *which are specialized to grow on particular substrates, have lost gustatory receptors and detoxification genes (*Drosophila* 12 Genomes Consortium et al. 2007; Dworkin and Jones 2009). Sometimes the losses are driven by positive selection, as has been suggested in the case of the *neverland *gene in *Drosophila pachea* (Lang et al. 2012) where positive selection appears to have favored a novel *neverland *allele that has lost the ability to metabolize cholesterol. In our study of gene families, the incompleteness of the annotation of *D. buzzatii* PCGs precludes us from being able to reliably identify gene families that lost family members.

To minimize the possibility of missing gene copies that were potentially collapsed into single genes during *D. buzzatii* genome assembly, we used sequence coverage to adjust the size of gene families. Two of the families that expanded as a result of this correction encoded chorion genes. However, chorion genes are known to undergo somatic amplifications in ovarian follicle cells (Claycomb and Orr-Weaver 2005), and the use of sequence coverage to correct for “missing” copies can be misleading in these cases. As there is no easy way to verify families that were placed into the expanded category due to high sequence coverage alone, our discussion below is limited to gene duplicates that were annotated in the *D. buzzatii* genome.

A recent survey of the functional roles of new genes across various taxa offers evidence for the rapid recruitment of new genes into gene networks underlying a wide range of phenotypes including reproduction, behavior, and development (Chen et al. 2013). A number of lineage-specific duplicates identified in our study fit this description, but further experimental confirmation of their functions through loss-of-function studies and characterization of molecular interactions are necessary. Among families that expanded in the *D. buzzatii*, the *D. mojavensis*, and the cactophilic lineages, 35% have functional annotations that are similar to those of rapidly evolving families identified in the analysis of the 12 *Drosophila* genomes (Hahn et al. 2007). These families include genes that are involved in proteolysis, zinc ion binding, chitin binding, sensory perception, immunity, and reproduction. A fraction of these expanded families may reflect physiological adaptations to a novel habitat. For example, given the importance of olfactory perception in recognition of the host cactus plants (Date et al. 2013), the duplication of an olfactory receptor in *D. buzzatii* may represent an adaptation to cactophilic substrates. Another *D. buzzatii *family includes *ninjurin*, a gene involved in tissue regeneration that is one of the components of the innate immune response (Boutros et al. 2002). In *D. mojavensis*, we also observe the duplication of an odorant receptor and, coinciding with a previous report (Croset et al. 2010), of an ionotropic glutamate receptor that belongs to a novel family of diversified chemosensory receptors (Benton et al. 2009; Croset et al. 2010). An aldehyde dehydrogenase is also duplicated in the *D. mojavensis* lineage and might reveal a role in detoxification of particular aldehydes and ethanol (Fry and Saweikis 2006). In the *D. buzzatii–D. mojavensis* lineage, one family contains proteins with the MD-2-related lipid recognition domain involved in pathogen recognition and in *D. mojavensis* we find a duplicate of a phagosome-associated peptide transporter that is involved in bacterial response in *D. melanogaster* (Charrière et al. 2010).

Several of the *D. mojavensis*-specific gene duplicates have been described as male and female reproductive proteins. Unlike the accessory gland proteins of *D. melanogaster*, the proteome of *D. mojavensis* accessory glands is rich in metabolic enzymes and nutrient transport proteins (Kelleher et al. 2009). Three of the expanded families include metabolic proteins previously identified as candidate seminal fluid proteins specific to *D. mojavensis* lineage (Kelleher et al. 2009). We also detect an increase of female reproductive tract proteases as a possible counter adaptation to fast-evolving male ejaculate (Kelleher and Markow 2009).

Three gene families are of particular interest among those that were expanded in the lineages leading to *D. buzzatii* and *D. mojavensis*, as they contain duplicates of genes with functions related to the regulation of JH levels. One family includes a new duplicate of JH esterase duplication gene (*Jhedup *in *D. melanogaster*). JH esterases are involved in JH degradation (Bloch et al. 2013), although *Jhedup *has much lower level of JH esterase activity than *Jhe* (Crone et al. 2007). Another family includes new duplicate that encodes protein with sequence similarity to hemolymph JH-binding protein (*CG5945* in *D. melanogaster*). JH-binding proteins belong to a large gene family regulated by circadian genes and affect circadian behavior, courtship behavior, metabolism, and aging (Vanaphan et al. 2012). This family includes JH-binding proteins that function as carriers of JH through the hemolymph to its target tissues (Bloch et al. 2013). The third family includes a new duplicate of a dopamine synthase gene (*ebony* in *D. melanogaster*). *ebony* is involved in the synthesis of dopamine, and it is known that dopamine levels affect behavior and circadian rhythms through regulation of hormone levels including JH (Rauschenbach et al. 2012). All three duplicates are expressed in *D. buzzatii *adults. At insect adult stage, JHs play a role in physiology and behavior, and their levels oscillate daily (Bloch et al. 2013). Gene duplications of JH-binding proteins (JHBP), JH esterases (JHE), and *ebony* may change the timing and levels of active JHs which, in turn, alter the behavior and physiology regulated by JHs. One interesting effect of mutations in circadian rhythm genes, or of direct perturbations of the circadian rhythm, is a reduced ethanol tolerance in *D. melanogaster* (Pohl et al. 2013). Intriguingly, *Jhedup *and another gene duplicated in the cactophilic lineage, *Sirt2* (a protein deacetylase), have been also shown to affect ethanol tolerance and sensitivity when mutated (Kong et al. 2010). Given that both *D. mojavensis* and *D. buzzatii* breed and feed on rotting fruit, a shift in tolerance to ethanol and other cactus-specific compounds is one of the expected adaptations associated with a switch to a cactus host. Future functional studies of these new duplicates are required to understand their role in physiological and behavioral changes associated with a change to a new habitat.

## Supplementary Material

Supplementary methods, figures S1–S6, and tables S1–S20 are available at *Genome Biology and Evolution* online (http://www.gbe.oxfordjournals.org/).

Supplementary Data
