# Supplementary material for: Genomics of Ecological Adaptation in Cactophilic Drosophila
Source: Genome Biol Evol. 2014 Dec 31;7(1):349–66. doi: 10.1093/gbe/evu291 (PMC4316639; doi:10.1093/gbe/evu291)
Supplement: Supplementary Data [file supp_7_1_349__index.html]

Genomics of ecological adaptation in cactophilic Drosophila — Genomics of ecological adaptation in cactophilic Drosophila — Genomics of Ecological Adaptation in Cactophilic Drosophila — Supplementary Data 

# Genomics of Ecological Adaptation in Cactophilic *Drosophila*

## Supplementary Data

files

**Files in this Data Supplement:**

- Supplementary Data - pdf file
- Supplementary Data - docx file
- Supplementary Data - docx file
- Supplementary Data - xls file
- Supplementary Data - docx file
- Supplementary Data - docx file
- Supplementary Data - xlsx file
