# Supplementary material for: Genomics of Ecological Adaptation in Cactophilic Drosophila
Source: Genome Biol Evol. 2014 Dec 31;7(1):349–66. doi: 10.1093/gbe/evu291 (PMC4316639; doi:10.1093/gbe/evu291)
Supplement: Supplementary Data [file supp_evu291_Supplementary_Figures_1-6.pdf]

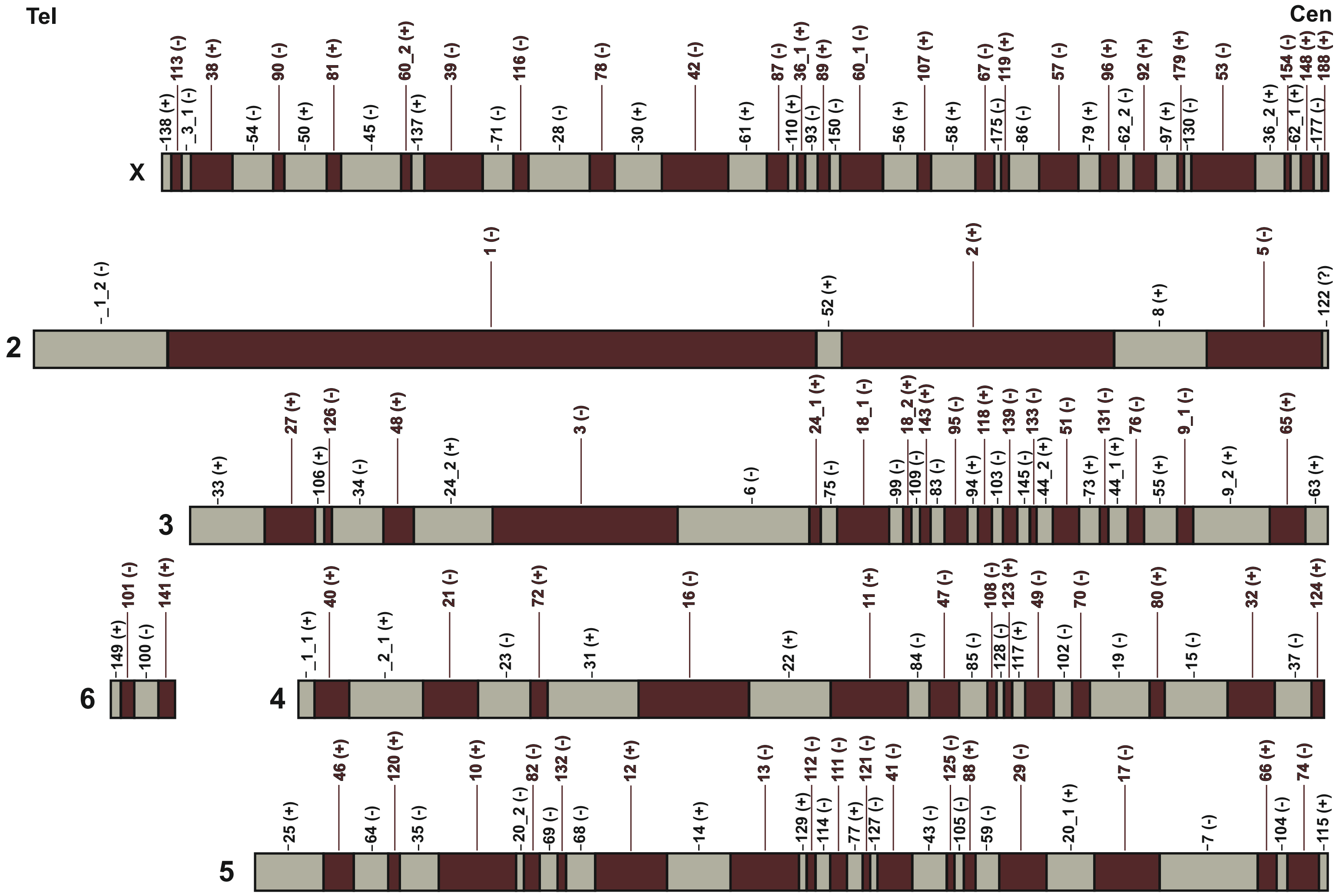

Tel
Cen
X
138 (+)
113 (-)
3_1 (-)
38 (+)
54 (-)
90 (-)
50 (+)
81 (+)
45 (-)
60_2 (+)
137 (+)
39 (-)
71 (-)
116 (-)
28 (-)
78 (-)
30 (+)
42 (-)
61 (+)
87 (-)
110 (+)
36_1 (+)
93 (-)
89 (+)
150 (-)
60_1 (-)
56 (+)
107 (+)
58 (+)
67 (-)
175 (-)
119 (+)
86 (-)
57 (-)
79 (+)
96 (+)
62_2 (-)
92 (+)
97 (+)
179 (+)
130 (-)
53 (-)
36_2 (+)
154 (-)
62_1 (+)
148 (+)
177 (-)
188 (+)
2
1_2 (-)
1 (-)
52 (+)
2 (+)
8 (+)
5 (-)
122 (?)
3
33 (+)
27 (+)
106 (+)
126 (-)
34 (-)
48 (+)
24_2 (+)
3 (-)
6 (-)
24_1 (+)
75 (-)
18_1 (-)
99 (-)
18_2 (+)
109 (-)
143 (+)
83 (-)
95 (-)
94 (+)
118 (+)
103 (-)
139 (-)
145 (-)
133 (-)
44_2 (+)
51 (-)
73 (+)
131 (-)
44_1 (+)
76 (-)
55 (+)
9_1 (-)
9_2 (+)
65 (+)
63 (+)
6
149 (+)
101 (-)
100 (-)
141 (+)
4
1_1 (+)
40 (+)
2_1 (+)
21 (-)
23 (-)
72 (+)
31 (+)
16 (-)
22 (+)
11 (+)
84 (-)
47 (-)
85 (-)
108 (-)
128 (-)
123 (+)
117 (+)
49 (-)
102 (-)
70 (-)
19 (-)
80 (+)
15 (-)
32 (+)
37 (-)
124 (+)
5
25 (+)
46 (+)
64 (-)
120 (+)
35 (-)
10 (+)
20_2 (-)
82 (-)
69 (-)
132 (-)
68 (-)
12 (+)
14 (+)
13 (-)
129 (+)
112 (-)
114 (-)
111 (-)
77 (+)
121 (-)
127 (-)
41 (-)
43 (-)
125 (-)
105 (-)
88 (+)
59 (-)
29 (-)
20_1 (+)
17 (-)
7 (-)
66 (+)
104 (-)
74 (-)
115 (+)

Figure S1. Order and orientation within *D. buzzatii* chromosomes of 158 scaffolds included in N90 index. The number of scaffolds in chromosomes X, 2, 3, 4, 5 and 6 is 48, 7, 38, 26, 35 and 4, respectively. Each scaffold is represented as a solid block and its orientation relative to telomere is marked by a positive (+) or negative (-) sign next to its identification number (? if direction is unknown).

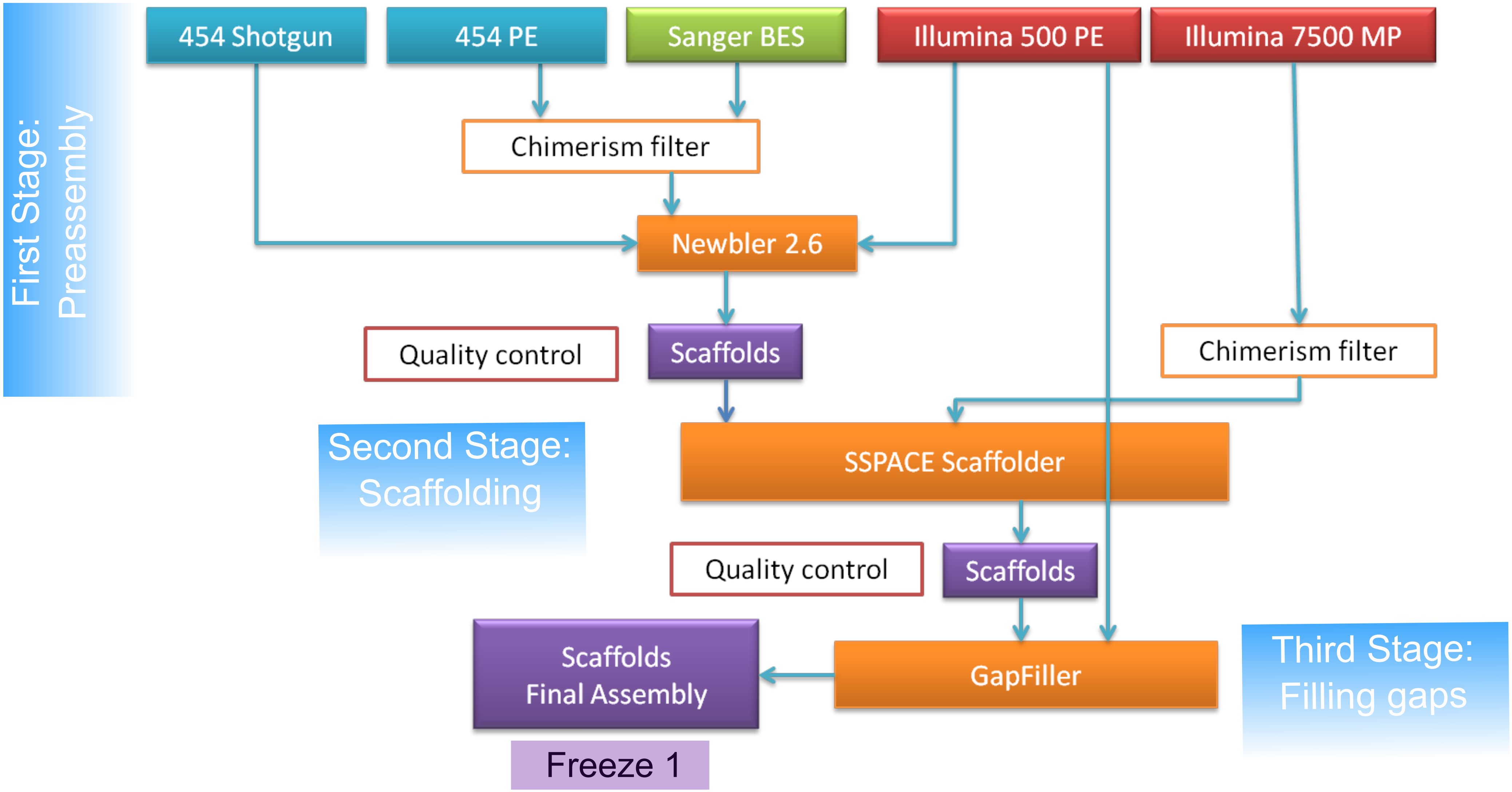

Figure S2. Assembly pipeline followed for st-1 *D. buzzatii* genome.

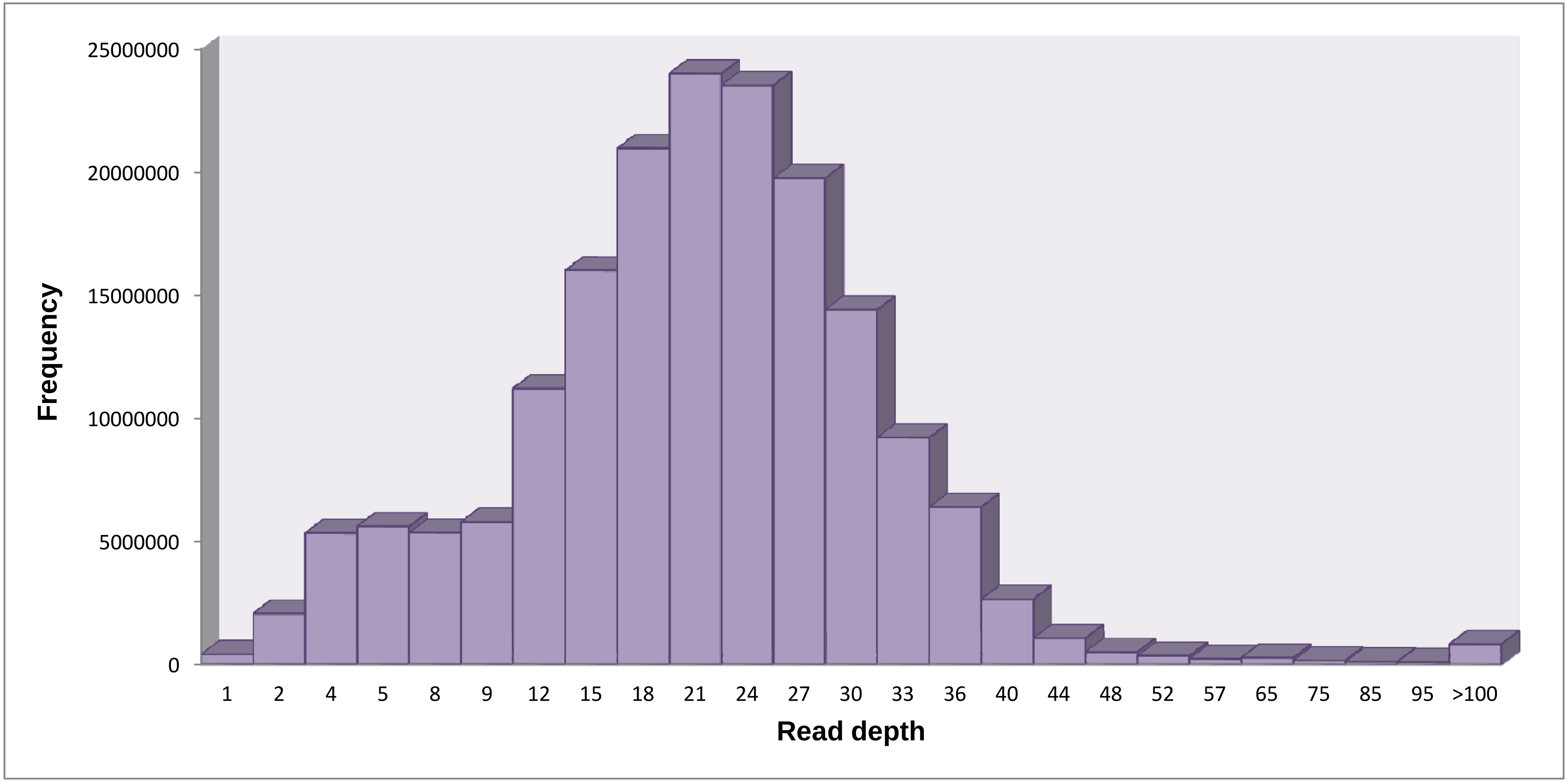

Figure S3. Read depth histogram of *D. buzzatii* preassembly.

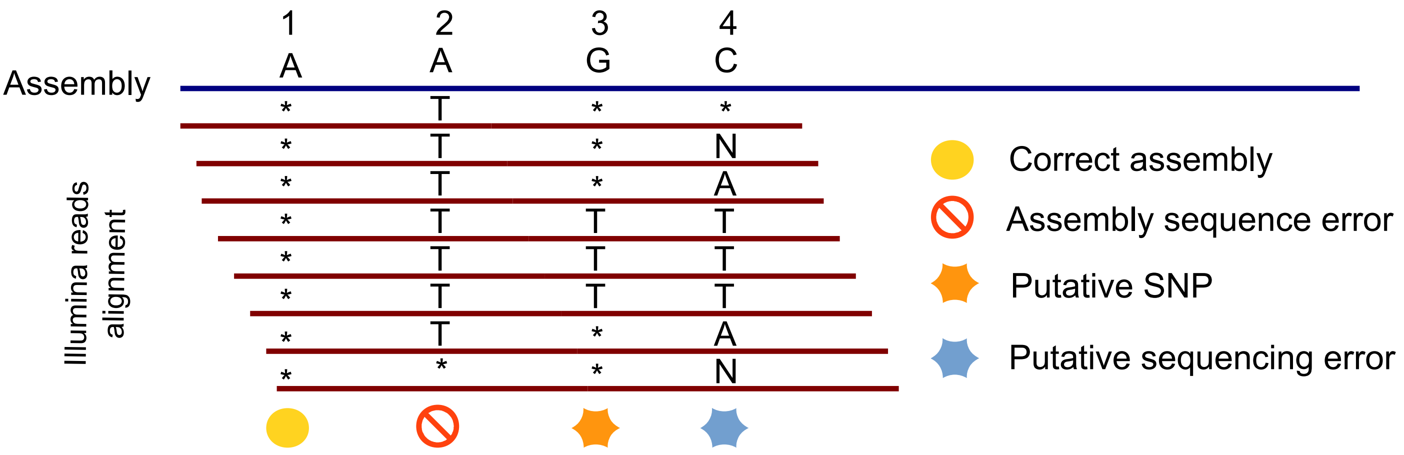

Position 1:
reads covering position = 8
#missmatches = 0
Error rate = 0/8 = 0

Position 2:
reads covering position = 8
#missmatches = 7
Error rate = 7/8 = 0,875 (>0.8 coverage)
>80% missmatches same base

Position 3:
reads covering position = 8
#missmatches = 3
Error rate = 3/8 = 0.345 (<0.8 coverage)

Position 4:
reads covering position = 8
#missmatches = 7
Error rate = 7/8 = 0.875 (>0.8coverage)
<80% missmatches same base

Figure S4. Algorithm designed to track putative sequence errors and polymorphic sites in freeze 1 assembly. Four different positions are described according to the results obtained by aligning Illumina reads. Positions with an error rate < 0.8 are considered correct positions (1). Positions in which more than 80% of the aligned reads having the same base do not match the assembly are pinpointing assembly errors (2). Polymorphic positions are detected if less than 80% but more than 20% of the aligned reads do not match the assembly and have the same base (3). Putative sequencing errors are detected when more than 80% of the bases do not match the assembly and they have random bases in the same position. This last category was not further analyzed.

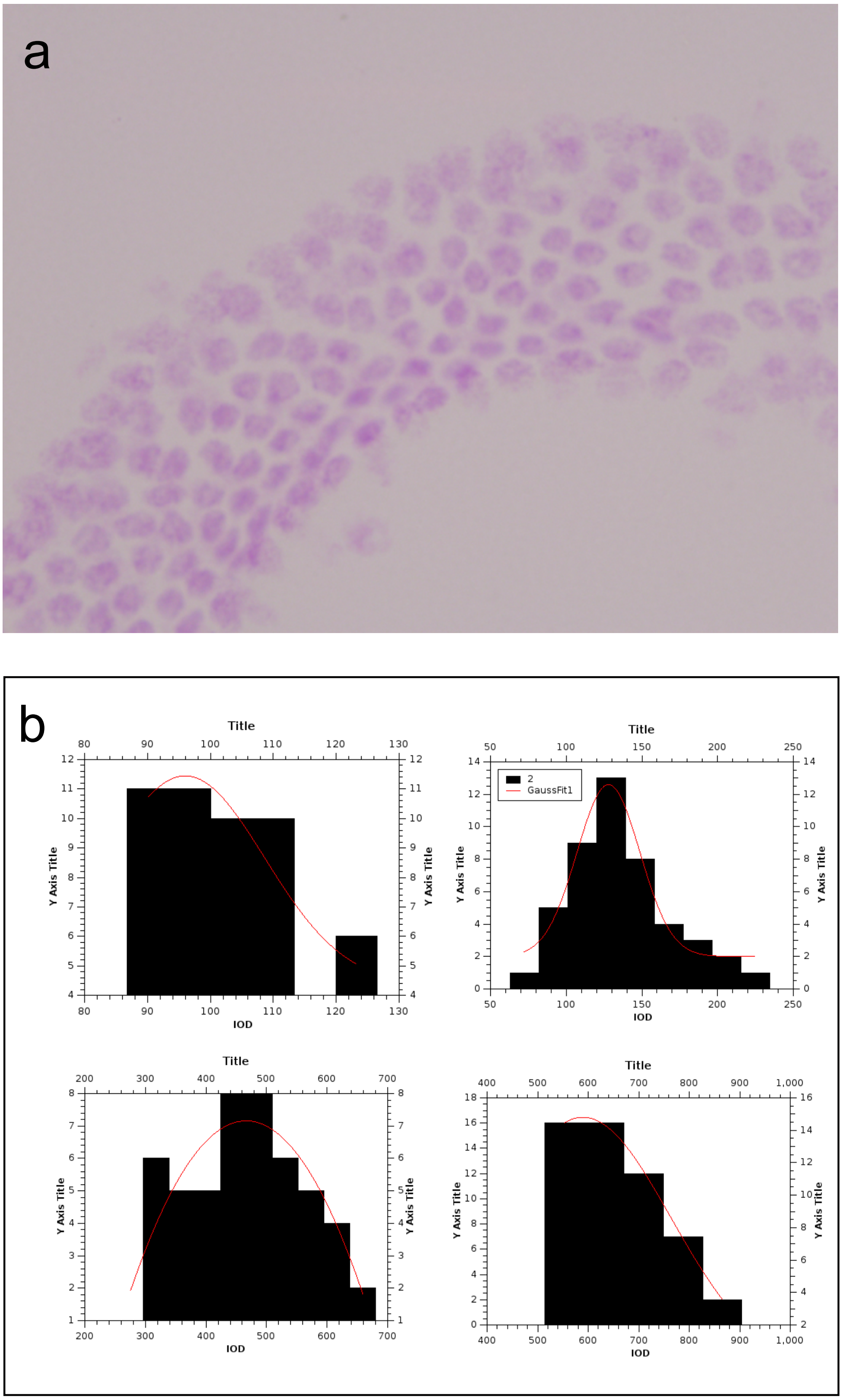

Figure S5. Genome size quantification of *D. buzzatii* st-1 and j-19 strains using IOD. Testicular cells analyzed from *D. buzzatii* st-1 strain (a) and normal distribution profiles that best fit to the IOD histogram representations (b). Fifty cells from each group were analyzed.

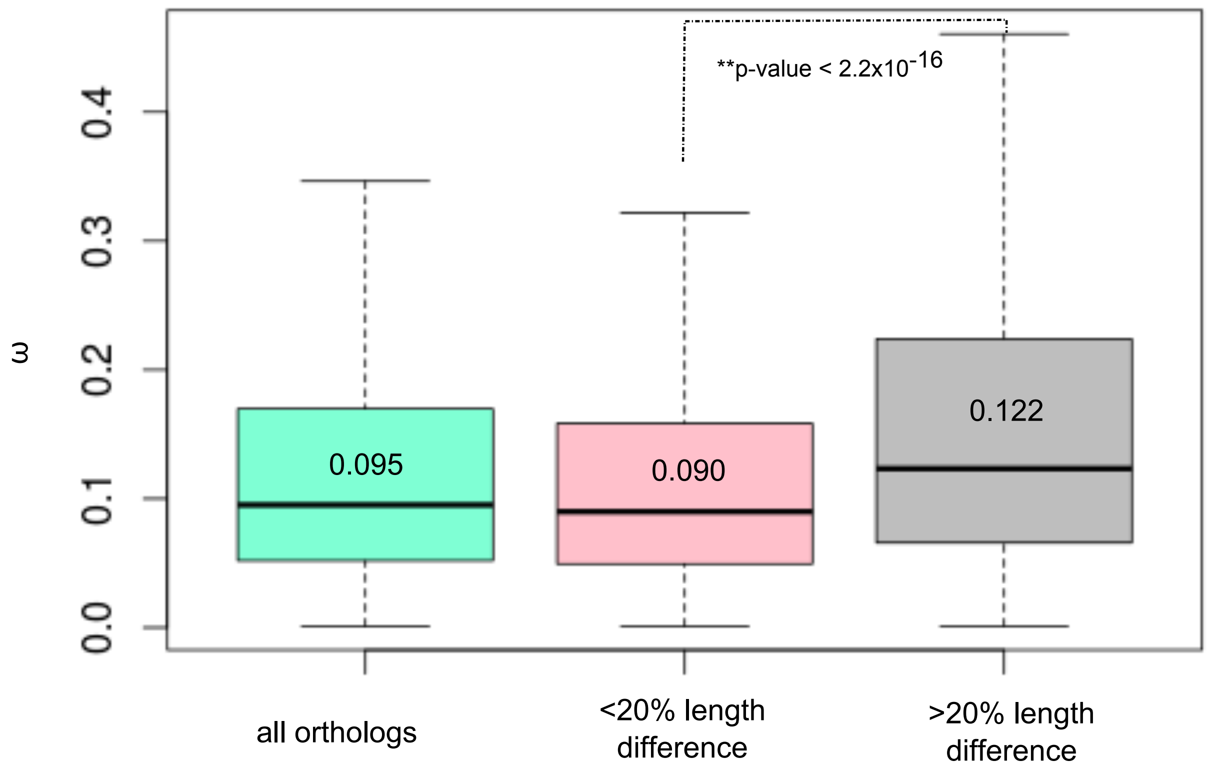

Figure S6. Distribution of dn/ds (=ω) for orthologs between *D. buzzatii* and *D. mojavensis*. Orthologous pairs that show a length difference higher than 20% increase the ω median of all gene set.
