# Supplementary material for: Genomics of Ecological Adaptation in Cactophilic Drosophila
Source: Genome Biol Evol. 2014 Dec 31;7(1):349–66. doi: 10.1093/gbe/evu291 (PMC4316639; doi:10.1093/gbe/evu291)
Supplement: Supplementary Data [file supp_evu291_Supplementary_Methods_R1.docx]

**SUPPLEMENTARY METHODS**

**Flies**

Two strains of *Drosophila buzzatii*, st-1 and j-19, were used. Strain st-1 was isolated from flies collected in Carboneras (Spain) by repeated sib-mating and selection for chromosome arrangement *2st* (Betrán et al. 1998). This strain is isogenic for the major part of chromosome 2 and highly inbred for the rest of the genome. Strain j-19 was isolated from flies collected in Ticucho (Argentina) using the balanced-lethal stock Antp/Δ^5^ (Piccinali et al. 2007). Individuals of j-19 strain are homozygous for chromosome arrangement *2j* (Cáceres et al. 2001).

**DNA extraction and sequencing**

DNA was extracted from male and female adults of strain st-1 using the sodium dodecyl sulfate (SDS) method (Milligan 1998) or the method described by Piñol et al. (1988) for isolating high molecular weight DNA. Reads from different sequencing platforms were generated for strain st-1 in order to achieve an accurate assembly of the genome of this strain (figure S2 and table S12). Shotgun reads (3 plates, ~8x) and paired-end (PE) reads (2 plates, ~3x) were generated using GS-FLX platform (454-Roche) at the Centre for Research in Agricultural Genomics (CRAG, Barcelona, Spain). PE reads were produced from three different libraries with inserts of 6 kb (one half-plate), 7 kb (one plate) and 8 kb (one half-plate). We removed duplicate reads from 454 sequences using CDHIT 3.1.2 (Li and Godzik 2006). We also generated ~100 bp PE reads (4 lanes, ~76x) from libraries with an insert size of ~500 bp using HiSeq2000 platform (Illumina) at the Centro Nacional de Análisis Genómico (CNAG, Barcelona, Spain). An accurate pipeline was designed in order to filter Illumina reads based on their length and quality. We first trimmed the read ends discarding bases with a quality lower than Q20 and then filtered low quality sequences (keeping only those with at least 95% of the bases with quality ≥ Q20). The final step was to discard exact duplicates and reverse complement exact duplicates from the final dataset. A mate pair (MP) library with ~7.5 kb fragments was also prepared and sequenced (one lane, ~12x) with Illumina at Macrogen Inc. (Seoul, Korea). Low quality reads as well as exact duplicates were removed (as before). Finally, we also used information provided by BAC end-sequences (BES) of 1,152 BAC clones covering *D. buzzatii* chromosome 2 (Guillén and Ruiz 2012).

***De novo* assembly**

The assembly of the genome of strain st-1 was performed in three stages (supplementary table S13). In the first stage, Newbler 2.6 was fed with filtered 454 reads (shotgun and PE), Sanger BES and one of the four Illumina PE lane to obtain an initial *de novo* preassembly (figure S2). Prior to the assembly, false or chimeric 454 PE reads were discarded by mapping all the paired sequences against the *D. mojavensis* masked genome (Drosophila 12 Genomes Consortium et al. 2007) using gsMapper (Newbler 2.6). Those reads coming from the same fragment that aligned to different chromosomes as well as those aligning to multiple locations in the *D. mojavensis* scaffolds were removed. Likewise, all BES were previously filtered by mapping them against the *D. mojavensis* genome in order to remove chimeric mates and artifacts using gsMapper. Out of the initial 2304 BES, 1799 reads were used for the preassembly. We used the “*heterozygotic mode”* option in Newbler 2.6 to allow for residual nucleotide variability in the inbreed st-1 strain. We also run the “*large or complex genome”* option as we were assembling a eukaryotic genome. Thus the assembly algorithm was prepared to deal with the problem of high-copy regions, although the number of output contigs was expected to be high. The preassembly contained 2,306 scaffolds. To estimate the number of chimeric artifacts, the 38 scaffolds contained in the N50 index were mapped to the *D. mojavensis* masked genome using NUCmer (Delcher et al. 2003). Three scaffolds that matched two or more regions located in different *D. mojavensis* chromosomes were considered chimeric and split.

In a second stage, Illumina MP reads were used by SSPACE (Boetzer et al. 2011) to link output >3kb scaffolds from the preassembly and obtain 815 larger scaffolds (supplementary table S13). A minimum number of three mate pairs were required to connect two sequences (k=3). Prior to this operation, all Illumina MP reads were mapped against the *D. buzzatii* contigs of the preassembly stage (table S13) using *bowtie2* (Langmead and Salzberg 2012)*.* We used only MP reads that obeyed the following criteria: (I) both end sequences from the same fragment mapped to different contigs (at unknown distance); and (II) both ends mapped in the same contig at a distance greater than 4.5 kb (thus excluding inward paired end contamination). SSPACE, the software used for the scaffolding step, excluded mates not mapping at the expected set distance. After this step, a second control for chimerism was performed (as before), detecting another three chimeric scaffolds (4, 26 and 98), which were split resulting in six new scaffolds.

The third stage consisted of filling the gaps (N's) using the three short PE Illumina libraries that were not included in the pre-assembly (supplementary table S13). GapFiller (Nadalin et al. 2012) was used in this stage, running 10 iterations and at least 4 reads needed to call a base during an extension (figure S2). To further control for chimerism, the 818 scaffolds in the N90 scaffold index resulting from the third assembly step were blasted against the *D. mojavensis* masked genome using MUMmer (Delcher et al. 2003) and the resulting hits were reordered according to the *D. mojavensis* coordinates. This method allowed the detection of inversion breakpoint regions shared by these two species and putative chimeric scaffolds. Under a conservative criterion, eight scaffolds (9, 18, 20, 24, 36, 44, 60, 62) mapping in more than one location in the same chromosome but in regions where no inversion breakpoints or other rearrangements were expected (see Results) were split. The final assembly, named Freeze 1, thus contains 826 scaffolds >3kb and N50 and N90 index are 30 and 158, respectively.

**Fold redundancy and base composition**

The distribution of read depth in the st-1 genome preassembly shows a Gaussian distribution with a prominent mode centered at ~22x (figure S3). Conceivably, the scaffolding and gap filling stages of the assembly did not alter significantly this distribution. However, its variance is much larger than that expected by random (~30 times higher), showing that there is an important bias on the coverage. In particular there is a long right tail that might reflect cases where highly similar repetitive sequences or duplicated genes were merged into the same consensus sequence. One such case of misassembly was observed in the Hsp68 genes. In most Drosophila genomes there are two almost identical Hsp68 gene copies arranged head-to-head (Guillén and Ruiz 2012). In the *D. buzzatii* genome only one copy was found but it was in the vicinity of a gap (filled with N’s) about the same size, suggesting that the assembler had merged all Hsp68 reads into a single gene leaving a gap in the place of the second copy.

Base composition of genes, exons and overall for Freeze 1 assembly is summarized in supplementary table S14. CG content is ~35% overall, ~42% in gene regions (including introns) and reaches ~52% in exons. Unidentified nucleotides (N’s) represent ~9% overall, ~4% in gene regions and 0.004% in exons. These patterns agree well with the reported higher CG content of genes and exons in many genomes including those of Drosophila (Adams et al. 2000; Heger and Ponting 2007; Díaz-Castillo and Golic 2007) and humans (Bulmer 1987; Lander et al. 2001).

**Sequence quality assessment and nucleotide polymorphism**

To assess the quality of the Freeze 1 assembly sequence, we used ~800 kb of Sanger sequences corresponding to five *D. buzzatii* BAC clones: 40C11 and 5H14 (Negre et al. 2005), 20O19 and 1N19 (Calvete et al. 2012) and 1B03 (Prada 2010). These BAC sequences were aligned against the genome sequence using MUMmer (Delcher et al. 2003). Some BAC regions containing repetitive elements matched multiple scaffold locations and were excluded (supplementary table S15). Considering only the unambiguously covered regions (97.6%), the genome sequence was 99.95% identical to that of the BAC sequences, giving an error rate of 0.0005 and a PHRED quality score of ~Q33.

In a second sequence quality assessment, we mapped the three Illumina runs (99,124,355 reads) that were used in the GapFiller stage of the assembly (figure S2) and RNA-Seq data from adult males (44,840,622 reads, see below) against the Freeze 1 assembly using bowtie2 (Langmead and Salzberg 2012). Mapping of genomic reads allowed us to assess the overall genome error rate, including both expressed and non-expressed regions, whereas mapping of RNA-Seq reads reported the error rate exclusively for expressed regions. We considered as assembly errors those positions where 80% or more of the reads did not match the genome base and at least 80% of these unmatched positions had the same nucleotide (figure S4). Under a conservative criterion the overall error rate was estimated to 0.0005 and the average quality ~Q33, as before. A similar value was estimated when aligning the RNA-Seq reads to the expressed regions of the genome (supplementary table S16).

Strain st-1 used for generating the *D. buzzatii* reference genome was isogenic for a large portion of chromosome 2 and highly inbreed for the remaining genome (see above). We estimated the amount of residual nucleotide polymorphism in this strain by aligning the Illumina reads against the genome Freeze 1 assembly (figure S4). An overall proportion of segregating sites of ~0.1% was estimated (supplementary table S17). About 15% of all the SNPs are located in gene sequences and 4% in coding exons. Thus the vast majority of SNPs are located in non-coding regions.

**Genome size estimation**

The genome size of two *D. buzzatii* strains, st-1 and j-19, was estimated by Feulgen Image Analysis Densitometry. The genome size of *D. mojavensis* 15081-1352.22 strain (193,826,310 bp) was used as reference (Drosophila 12 Genomes Consortium et al. 2007). Testes from anesthetized males of both species and strains were dissected in saline solution and fixed in acetic-alcohol 3:1. Double preparations of *D. mojavensis* and *D. buzzatii* were prepared by crushing the fixed testes in 50% acetic acid. Following Ruiz-Ruano et al. (2011), the samples were stained by Feulgen reaction including a 5N HCl incubation for 5 minutes. Images obtained by optical microscopy were analyzed with the pyFIA software (Ruiz-Ruano et al. 2011) (figure S5 and supplementary table S18).

**Chromosome organization and evolution**

The 826 scaffolds in Freeze 1 were assigned to chromosomes by aligning their sequences with the *D. mojavensis* genome using blastn from MUMmer (Delcher et al. 2003). In addition, the 158 scaffolds in the N90 index were mapped, ordered and oriented in the chromosomes (figure S1). The seven scaffolds corresponding to chromosome 2 were ordered and oriented using *D. buzzatii* BAC-based physical map and BAC-end sequences (González et al. 2005, Guillén and Ruiz 2012). Those scaffolds mapping to chromosomes X, 4, 5 and 6 were ordered and oriented by conserved linkage (Schaeffer et al. 2008). Briefly, we looked for the position in *D. mojavensis* of genes located at the ends of *D. buzzatii* scaffolds. When two of these genes are closely located in the *D. mojavensis* genome (<200 kb in most cases) we can infer that they are also close in *D. buzzatii*, assuming synteny conservation, and then the respective scaffolds must be adjacent. This method works as far as there are no inversion breakpoints between the two scaffolds and gave consistent results for the four forementioned chromosomes. In contrast, for chromosome 3, it yielded ambiguous or inconsistent results. We had to resort to *in situ* hybridization of PCR generated probes to anchor chromosome 3 scaffolds to *D. buzzatii* polytene chromosomes (Delprat et al. in preparation).

In order to determine the organization of the HOX gene complex (HOM-C), the eight Drosophila HOX genes were searched bioinformatically in the *D. buzzatii* genome and found in three chromosome 2 scaffolds: 2, 5 and 229. Scaffold 2 contained four Hox genes (*pb, Scr, Antp* and *Ubx*) and scaffold 5 another three (*lab, abdA* and *AbdB*) (see Results). The eighth HOX gene, *Dfd*, was found in the small scaffold 229 (49,930 bp). We looked for the genomic position of this scaffold using BAC-end sequences and found that those of three BACs (3A12, 9B20 and 25B04) anchored this scaffold inside scaffold 2, precisely within the HOX gene complex where there is a 65-kb gap filled with N’s. We concluded that this was a case of misassembly and the correct order of *D. buzzatii* HOX genes at this chromosomal site must be *pb, Dfd, Scr, Antp* and *Ubx.* All genes (HOX genes, HOX-derived genes and non-HOX genes) within the HOM-C were manually annotated using the available information (Negre et al. 2005), the annotated *D. mojavensis* and *D. melanogaster* genomes, and the RNA-seq data generated for *D. buzzatii*.

**Repeat identification and masking**

A library of transposable elements (TEs) was constructed combining three different collections of repeats. The first collection was compiled blasting FlyBase canonical set of TEs against an early assembly of *D. buzzatii* genome. For each query several significant hits were manually inspected in order to recover the most complete TE copy. The second collection was built with RepeatScout 1.0.5 (Price et al. 2005) and classified by Repclass (Feschotte et al. 2009) and the third is the result of RepeatModeler 1.0.5 (Smit and Hubley 2008), with RepeatScout and RECON (Bao and Eddy 2002), both using the *D. buzzatii* early assembly. Manual analyses to reduce redundancy and remove possible protein coding genes were performed with RepeatMasker and blast searches resulting in a library with 357 TE sequences. This library was used to mask the repeats from Freeze 1 assembly with RepeatMasker v3.2.9 (Smit et al. 1996) and then annotate the protein coding genes (see below).

A second and more comprehensive TE library (4,802 sequences) was generated adding Repbase (Jurka et al. 2005) repeats from *Insecta* species to the previous library and running again RepeatScout and RepeatModeler with *D. buzzatii* Freeze 1 assembly. Additionally, sequences classified as simple repeats, satellite or low complexity, were removed from the library. Finally, a blast analysis was performed to filter non-TE related sequences. Sequences with significant hits (e-value<1e-25) to *D. mojavensis* CDS and at the same time with no significant similarity to repeats deposited in Repbase were removed. This second TE library was then used to annotate and classify *D. buzzatii* TEs running RepeatMasker with the following options cutoff 250, -nolow and –norna, to prevent masking any low complexity regions and small RNA genes.

In order to identify satDNAs (highly abundant tandemly repeated DNA motifs) from the genome of *D. buzzatii*, we used the Tandem Repeats Finder (TRF) software (version 4.04) (Benson 1999). Tandem repeats searches were performed in all contigs using the command line version of TRF with parameters 1, 1, 2, 80, 5, 200 and 750 for *match*, *mismatch*, *indel*, *probability of match*, *probability of* *indel*, *min. score* and *max. period*, respectively. Repeats with less than 50 bp were eliminated from the dataset. We developed a series of scripts and pipelines for clustering similar tandem repeats into major families and to eliminate redundancy between families (de Lima et al. in preparation). The outcome produced a table containing the repeat size, consensus sequence and genomic fraction of every tandem repeat family identified. From the final collection of tandem repeats, we selected the most likely satDNA families based on three main parameters: (i) abundance; (ii) no sequence similarity with transposable elements or to other non-satellite genomic elements (inferred by screening the Repbase, Genbank and FlyBase databases) and (iii) the presence of several contigs made exclusively by repeats from the same tandem repeat family.

**Developmental transcriptome**

Flies of the *D. buzzatii* st-1 strain were reared on standard cornmeal-yeast-agar culture media. Ten to twenty individuals from each of five different life stages (embryos, larvae, pupae, adult males and adult females) were collected and frozen at -80ºC. RNA from frozen samples was processed using the TruSeq RNA sample preparation kit provided by Illumina. The protocol included a poly-A selection to enrich for mRNA. Library preparation was carried out at Cornell's Molecular Biology and Genetics Department, whereas RNA sequencing was done at Weill Cornell Medical College. The average insert size of the libraries from the 5 samples was 264 bp. Sequencing at PE 100 bp was performed on a Hi-Seq2000 Illumina Sequencer. A total of 378,647,052 raw reads were generated (38 Gb of sequence) comprising between 60 and 89 million reads from each of the 5 samples. RNA-Seq reads were trimmed and filtered by quality (at least 95% of the bases had a quality ≥ Q20) (supplementary table S19). Filtered reads were mapped to Freeze 1 masked genome using TopHat version 1.3.3 allowing only for uniquely mapped reads (Trapnell et al. 2009). The common setting parameters used among different stages were: -g 1 (maximum multihits) -F 0 (suppression of transcripts below this abundance level) and -i 40 (minimum intron length). The rest of parameters were set by default.

We run Cufflinks to reconstruct transcripts models and their expression level for each stage (Trapnell et al. 2010) using Annotation Release 1 as reference (-g option activated). This allowed us to identify new isoforms from expressed protein-coding genes (PCG) and also non-coding RNA (ncRNA) genes. Transcription levels along the genome sequence and transcripts inferred by Cufflinks for each stage are included in the genome browser of the *D. buzzatii* Genome Project web ([http://dbuz.uab.cat](http://dbuz.uab.cat/)).

**Protein-coding gene annotation**

The masked Freeze 1 assembly was used to annotate PCGs using a strategy that combined both *ab initio* and homology-based predictions. We used two HMM-based algorithms, Augustus (Stanke and Waack 2003) and SNAP (Korf 2004), and a dual-genome *de novo* software, N-SCAN (Korf et al. 2001) using as guide the alignment between *D. buzzatii* Freeze 1 assembly and *D. mojavensis* masked genome (release 1.3). Exonerate was run to identify conserved genes aligning both *D. mojavensis* and *D. melanogaster* protein databases to Freeze 1 assembly (Slater and Birney 2005). All these predictions were combined by a weight-based consensus generator, EVidence Modeler (EVM) (Haas et al. 2008) using the following weights: Exonerate *D. mojavensis* (9), Exonerate *D. melanogaster (6),* NSCAN (6), Augustus (2) and SNAP (2). The EVM gene set contained 12,102 gene models.

There were 1,555 genes annotated by Exonerate but not reported by EVM due to their structural properties. We included these genes in Annotation Release 1 by combining EVM and Exonerate annotations using mergeBed tool from Bedtools package (Quinlan and Hall 2010). The Annotation Release 1 includes 13,657 annotated genes (12,102 annotated by EVM and 1,555 genes detected only by Exonerate). The 1,555 genes annotated only by exonerate were shorter (Wilcoxon test, W=81226636, p-value<2.2e-16) and had fewer exons (W=15142546, p-value<2.2e-16). This fact indicates that algorithms that annotate genes by generating a consensus from multiple evidences are not efficient at identifying short and monoexonic genes. Some genes from the Annotation Release 1 contain internal stop codons and/or lack stop or start codons suggesting they might be misannotated PCG or pseudogenes (supplementary table S20). We filtered those PCG that show at least one internal stop codon and/or were not multiple of three, leaving a total of 12,977 high-confidence protein-coding sequences for further analyses.

We computed the number of wrong assembled positions contained in the total span of the gene models as well as the errors located within exons of Annotation Release 1 (see above). The vast majority of genes (91.3%) and exon (99.2%) sequences showed no error nucleotides. Thus, we concluded that errors are mainly contained in non-exonic regions, and both the detection of positive selection and the divergence pattern analyses carried out subsequently will not be significantly altered by misassembled sequences (Schneider et al. 2009).

**Detection of genes under positive selection**

To test for positive selection we made a comparison between different pairs of codon substitution models. We first estimated the dn/ds ratios of 11,154 orthologs between *D. mojavensis* and *D. buzzatii*. Orthologus pairs that showed a length difference higher than 20% were excluded, as well as those orthologs with a ds >1, leaving a total of 9,114 PCGs. Then we run two site models on this gene set: M7 (beta), which does not allow for positively selected sites (ω>1), and M8 (beta&ω), which includes one extra class of sites to the beta model allowing for sites with ω>1 (Yang 2007). Both models were then compared using a likelihood-ratio test (LRT). We also run two more site models, M1a and M2a, and compared them again using the LRT test. Only genes that were detected as putatively under positive selection by both model comparisons were analyzed in further detail (see Results).

To perform the branch-site tests of positive selection, we identified 8,328 1:1:1:1 orthologs among the four available Drosophila subgenus species: *D. buzzatii*, *D. mojavensis*, *D. virilis* and *D. grimshawi* using OrthoDB version 6 database (Kriventseva et al. 2008). Branch-site models allow us detecting positive selection that affects particular sites and branches of the phylogeny. We decided to test for positive selection on three different lineages: *D. mojavensis* lineage, *D. buzzatii* lineage, and the lineage that led to the two cactophilic species (*D. buzzatii* and *D. mojavensis*) (supplementary table S3). We run Venny software (Oliveros 2007) to create a Venn diagram showing shared selected genes among the different models. Gene expression information for positively selected genes was extracted from the Cufflinks output (see above).

**Detection of orphan genes**

We identified genes that are only present in the two cactophilic species, *D. mojavensis* and *D. buzzatii*, by blasting the amino acid sequences from the 9,114 1:1 orthologs between *D. mojavensis* and *D. buzzatii* against all the proteins from the remaining 11 Drosophila species available in FlyBase protein database (excluding *D. mojavensis*). Proteins that showed no similarity with any Drosophila known gene product were considered putative orphans. We used a cutoff value of 1e-05 to avoid spurious hits. From the initial single-copy orthologs set between *D. mojavensis* and *D. buzzatii*, 117 proteins showed no similarity with any predicted Drosophila polypeptides. We used this set to study genes unique to the cactophilic lineage (Supplementary table S3) and analyzed their expression pattern with TopHat and Cufflinks (see above).

**Gene Duplication Analyses**

The longest isoforms of annotated PCGs from the four species of the Drosophila subgenus (supplementary table S4) were used for the analysis of gene duplications in cactophilic species (*D. mojavensis* and *D. buzzatii*) and in the lineage leading to *D. buzzatii*. We ran all-against-all blastp (version 2.2.25+; Altschul et al. 1997) and selected hits with alignment length extending over at least 50% of both proteins and with amino acid identity of at least 50%. These proteins were clustered using Markov Cluster Algorithm (Enright et al. 2002) with the bit score value as the similarity measure and an inflation parameter (I) of 2. We then removed 636 genes that matched transposable elements (supplementary table S5) that were identified by tBlastn with an E-value cut off 10E-20 against the TE library consisting of Repbase Hexapoda TE set (Jurka et al. 2005) and the newly-identified *D. buzzatii* TEs (this work). We also removed 664 *D. buzzatii* ORFs that contained internal stop codons (supplementary table S6). To account for possible missing gene copies that might have been collapsed during the assembly of *D. buzzatii* genome an additional gene copy was added to a gene family when gene sequence coverage in *D. buzzatii* exceeded 2X the average coverage. This correction added a total of 155 genes, increasing the number of family members in 64 gene families. The final dataset included a total of 56,587 proteins from the 4 species clustered into 19,567 families (supplementary table S7)

Gene counts for each family from the 4 species were analyzed with an updated version of CAFE (CAFE 3.1 provided by the authors; Han et al. 2013) to identify lineage-specific expansions. Given the phylogeny and gene family sizes in each species, CAFE estimates the maximum likelihood value of gene birth and death rates and infers the ancestral family sizes. With known family sizes at the tree nodes, families that expand or contract in a particular lineage can be identified. This analysis was performed only with families for which at least one member is inferred to be present at the root of the tree. Our detection of expanded families was based on the 2-parameter model (separate birth rates for *D. buzzatii* branch and the rest of the tree) because it was significantly better than a single-parameter model (p<10-4; likelihood ratio test).

The sets of CAFE-identified expanded families in the *D. buzzatii* (86 families) and *D. mojavensis* (127 families) genomes were examined for the presence of lineage-specific duplications. First, we used PAML package (Yang 2007) to calculate pairwise dS for all family members and narrowed the list of candidates by selecting families that included members with dS<0.4 (30 families in *D. buzzatii* and 86 families in *D. mojavensis*). These families were further examined manually and lineage-specific duplications were inferred when no hits were found in the syntenic region of the genome with a missing copy (20 families in *D. buzzatii* and 17 families in *D. mojavensis*). The syntenic regions were identified by the closest *D. mojavensis* – *D. buzzatii* orthologs of the genes flanking the new duplicate in the *D. buzzatii* or *D. mojavensis* genome. *D. buzzatii*-specific RNA-mediated duplications were identified by examining intron-less and intron-containing gene family members. A duplicate was considered a retrocopy if its sequence spanned all introns of the parental gene.

The number of families identified by CAFE as expanded along the cactophilic lineage was reduced by considering only those families that were also found in expanded category after rerunning the analysis with a less stringent cutoff (35% amino acid identity, 50% coverage). This procedure eliminates the effects of our arbitrary threshold in gene family assignment. The overlapping set of expanded families (27 families) was manually examined to verify the absence of *D. buzzatii* and *D. mojavensis* new family members in the *D. virilis* genome (confirmed in 20 families).

Functional annotation (i.e., GO term) for all expanded families was obtained using the DAVID annotation tool (Huang et al. 2009a; Huang et al. 2009b). For genes without functional annotation in DAVID, annotations of *D. melanogaster* orthologs were used. Consensus annotation for each expanded family is provided in supplementary table S11.

**Additional References**

Altschul SF et al. 1997. Gapped BLAST and PSI-BLAST: a new generation of protein database search programs. Nucleic Acids Res. 25:3389-402

Bao Z, Eddy SR. 2002. Automated *de novo* identification of repeat sequence families in sequenced genomes. Genome Res. 12: 1269–1276.

Bulmer M. 1987. A statistical analysis of nucleotide sequences of introns and exons in human genes. Mol Biol Evol. 4: 395–405.

Cáceres M, Puig M, Ruiz A. 2001. Molecular characterization of two natural hotspots in the *Drosophila buzzatii* genome induced by transposon insertions. Genome Res. 11: 1353–1364.

Díaz-Castillo C, Golic KG. 2007. Evolution of gene sequence in response to chromosomal location. Genetics 177: 359–374.

Feschotte C, Keswani U, Ranganathan N, Guibotsy ML, Levine D. 2009. Exploring repetitive DNA landscapes using REPCLASS, a tool that automates the classification of transposable elements in eukaryotic genomes. Genome Biol Evol. 1: 205–220.

Heger A, Ponting CP. 2007. Evolutionary rate analyses of orthologs and paralogs from 12 Drosophila genomes. Genome Res. 17: 1837–1849.

Lander ES, et al. 2001. Initial sequencing and analysis of the human genome. Nature 409: 860–921.

Langmead B, Salzberg SL. 2012. Fast gapped-read alignment with Bowtie 2. Nat Methods 9: 357–359.

Li W, Godzik A. 2006. Cd-hit: a fast program for clustering and comparing large sets of protein or nucleotide sequences. Bioinformatics 22: 1658–1659.

Piccinali R, Mascord L, Barker J, Oakeshott J, Hasson E. 2007. Molecular Population Genetics of the α-Esterase5 Gene Locus in Original and Colonized Populations of Drosophila buzzatii and Its Sibling Drosophila koepferae. J Mol Evol. 64: 158–170.

Price AL, Jones NC, Pevzner PA. 2005. *De novo* identification of repeat families in large genomes. Bioinformatics 21 Suppl 1: i351–358.

Quinlan AR, Hall IM. 2010. BEDTools: a flexible suite of utilities for comparing genomic features. Bioinformatics 26: 841–842.

Smit AFA, Hubley R, Green P. *RepeatMasker Open-3.0*. 1996-2010. <http://www.repeatmasker.org>.

Smit AFA, Hubley R. *RepeatModeler Open-1.0*. 2008-2010. <http://www.repeatmasker.org>.
