# Supplementary material for: Genomics of Ecological Adaptation in Cactophilic Drosophila
Source: Genome Biol Evol. 2014 Dec 31;7(1):349–66. doi: 10.1093/gbe/evu291 (PMC4316639; doi:10.1093/gbe/evu291)
Supplement: Supplementary Data [file supp_evu291_Supplementary_Tables_S12-S20.docx]

**Table S12. Summary of sequencing data.**

| Strain | Platform | Library | | Mean  insert size  (kb) | #Raw reads | #Filtered reads | Mean read length (bp) | Expected coverage |
| --- | --- | --- | --- | --- | --- | --- | --- | --- |
|  |  | Type | # plates (454)  or lanes (Illumina) |  |  |  |  |  |
| st-1 | 454 | Shotgun | 3 | - | 4219296 | 3857039 | 335.23 | 8x |
|  |  | PE | 2 | 6-8 | 2501837 | 1691215 | 304.92 | 3x |
|  | Sanger | BES | - | 150 | 2304 | 1799 | 698.2 | ~0.01x |
|  | Illumina | PE | 4 | 0.5 | 447062156 | 114499279 | 106.3 | 76x |
|  |  | MP | 1 | 7.5 | 41846306 | 19292893 | 97.8 | 12x |

**Table S13. Three stages in the assembly of *D. buzzatii* st-1 genome.**

| Stage | Input | #  Scaffolds  (> 3 kb) | # putative chimerics (split) | N50 scaffold index | Max scaffold size | #N's | #gaps |
| --- | --- | --- | --- | --- | --- | --- | --- |
| De novo Pre-assembly (Newbler) | All 454 + BES + 1 library Illumina short PE | 2306 | 3 (interchromosomal) | 38 | 14579794 | 18060254 | - |
| Scaffolding (SSPACE) | Pre-assembled scaffolds + MP libray | 815 | 3 (interchromosomal) | 29 | 16289485 | 18991294 | 13409 |
| Gapfilling (GapFiller) | Scaffolds + 3 Illumina short PE | 818 | 8 (intrachromosomal) | 30 | 16306990 | 14974169 | 11462 |

**Table S14. Base composition by genome features.**

| Base  composition | Genome | Genes | Exons |
| --- | --- | --- | --- |
| AT | 55.81 % | 54.24 % | 48.17 % |
| GC | 34.92 % | 42.00 % | 51.83 % |
| N | 9.27 % | 3.76 % | 0.004 % |
| Total bases | 161490851 | 42433860 | 20364820 |
| Fraction | 100 % | 26.28 % | 12.61 % |

**Table S15. Quality control of Freeze 1 assembly using sequenced BACs.**

| BAC | Chromosome | Length (bp) | Unambiguous bp covered (%) | Average identity  (%) | Matched scaffolds | | |
| --- | --- | --- | --- | --- | --- | --- | --- |
|  |  |  |  |  | Number of scaffolds | Freeze 1 scaffold id. | Aligned blocks |
| 1B03 | 2 | 258840 | 97.29 | 99.96 | 1 | scaffold1 | 8 |
| 1N19 | 2 | 138724 | 98.97 | 99.92 | 1 | scaffold1 | 8 |
| 20O19 | 2 | 143293 | 98.24 | 100 | 1 | scaffold1 | 5 |
| 40C11 | 2 | 132938 | 100.00 | 99.88 | 1 | scaffold2 | 6 |
| 5H14 | 2 | 124024 | 93.31 | 99.97 | 1 | scaffold5 | 12 |

**Table S16. Assembly error rate inferred by mapping genomic and RNAseq reads to Freeze 1 sequence. The overall error rate was computed using a coverage threshold of 4 aligned reads per position.**

|  | Genomic reads mapping | | RNAseq male adults reads mapping | |
| --- | --- | --- | --- | --- |
|  | # Putative assembly sequence errors | Error rate | # Putative assembly sequence errors | Error rate |
| No coverage threshold | 182598 | 0.00125 | 71499 | 0.00153 |
| Coverage threshold ≥4 | 68898 | 0.00047 | 19042 | 0.00062 |

**Table S17. Polymorphism rate estimation by mapping Illumina reads to Freeze 1 assembly.**

|  | Gapfiller reads mapping | |
| --- | --- | --- |
|  | # Polymorphic positions | Polymorphism rate |
| No coverage threshold | 148772 | 0.00102 |
| Coverage threshold ≥4 | 141648 | 0.000972 |

**Table S18. Optical Density (IOD) and genome size estimation.**

|  | IOD | | Genome size (pg) | | Genome size (Mb) | |
| --- | --- | --- | --- | --- | --- | --- |
| Species | j19 | st1 | j19 | st1 | j19 | st1 |
| *D. buzzatii* | 96.56 | 467.03 | 0.149 | 0.156 | 146 | 153 |
| *D. mojavensis* | 128.27 | 591.20 | 0.198^a^ | 0.198^a^ | 194^b^ | 194^b^ |

^a^ Estimated by dividing genome size in Mb by 978 Mb/pg.

^b^ Total assembly size (Drosophila 12 Genomes Consortium).

**Table S19. RNAseq reads per sample**

| Sample | Yield (Mb) | Reads  (x 10^6^) | % bp Q ≥ 30 | Mean Quality Score | Paired filtered reads (x 10^6^) | Reads used by TopHat  (x 10^6^) | Reads yielding unique hits  (x 10^6^) |
| --- | --- | --- | --- | --- | --- | --- | --- |
| Embryos | 9051 | 89.6 | 87.05 | 34.26 | 68.5 | 68.4 | 50.9 |
| Larvae | 6084 | 60.2 | 87.51 | 34.42 | 46.5 | 46.4 | 30.3 |
| Pupae | 7070 | 69.9 | 86.13 | 33.94 | 52.4 | 52.4 | 45.8 |
| Female adults | 8658 | 85.7 | 85.77 | 33.85 | 63.6 | 63.6 | 55.8 |
| Male adults | 7382 | 73.1 | 87.03 | 34.25 | 55.9 | 55.8 | 44.8 |
| Total | 38245 | 378.5 | 86.70 | 34.14 | 286.9 | 286.6 | 227.6 |

**Table S20. Features of PCG models in Annotation Release 1.**

|  | EVM | Exonerate | Total |
| --- | --- | --- | --- |
| Annotated PCGs | 12102 | 1555 | 13657 |
| Putatively correct CDS | 11213 | 0 | 11213 |
| CDS with internal stop codons | 334 | 330 | 664 |
| CDS lacking start codon | 163 | 0 | 163 |
| CDS lacking stop codon | 308 | 654 | 962 |
| CDS lacking start and stop codons | 68 | 571 | 639 |
| CDS not multiple of 3 | 16 | 0 | 16 |
