# Supplementary material for: Genomics of Ecological Adaptation in Cactophilic Drosophila
Source: Genome Biol Evol. 2014 Dec 31;7(1):349–66. doi: 10.1093/gbe/evu291 (PMC4316639; doi:10.1093/gbe/evu291)
Supplement: Supplementary Data [file supp_evu291_Supplementary_Table_S1.docx]

**Table S1. Protein-coding gene content of *D. buzzatii* genome compared to those of *D. mojavensis* and *D. melanogaster*.**

| Species | *D. buzzatii* | *D. mojavensis R1.3* | *D. melanogaster*  *R5.55* |
| --- | --- | --- | --- |
| Number of genes | 13657 | 14595 | 13937 |
| Mean gene size (bp) | 3108 | 4429 | 6656 |
| Mean protein size (aa) | 498 | 494 | 690 |
| Longest gene size (bp) | 67103 | 299059 | 396068 |
| Shortest gene size (bp) | 63 | 105 | 117 |
| Longest protein size (aa) | 14469 | 8926 | 22949 |
| Shortest protein size (aa) | 21 | 34 | 11 |
| Mean number of exons | 3.80 | 3.78 | 5.50 |
