# Supplementary material for: Genomics of Ecological Adaptation in Cactophilic Drosophila
Source: Genome Biol Evol. 2014 Dec 31;7(1):349–66. doi: 10.1093/gbe/evu291 (PMC4316639; doi:10.1093/gbe/evu291)
Supplement: Supplementary Data [file supp_evu291_Supplementary_Table_S2.docx]

**Supplementary Table S2**

**(A)** Number of protein coding genes (PCG) and non-coding genes (ncRNA) expressed along *D. buzzatii* development.

| Stage | PCG | ncRNA | Total |
| --- | --- | --- | --- |
| Embryos | 8552 | 1208 | 9760 |
| Larvae | 8709 | 810 | 9519 |
| Pupae | 10485 | 1574 | 12059 |
| Adult females | 9310 | 1037 | 10347 |
| Adult males | 10347 | 1824 | 12171 |
| Total | **47403** | **6453** | **53856** |

**(B)** Number of PCG and ncRNA genes expressed in one or more stages.

| Stages | PCG | ncRNA | Total |
| --- | --- | --- | --- |
| 1 | 925 | 1292 | 2217 |
| 2 | 1655 | 689 | 2344 |
| 3 | 1322 | 393 | 1715 |
| 4 | 1618 | 326 | 1944 |
| 5 | 6546 | 260 | 6806 |
| Total | **12066** | **2960** | **15026** |

**(C)** Distribution of putatively selected genes expressed along *D. buzzatii* development.

| Stage | Selected | Non- selected | Total |
| --- | --- | --- | --- |
| Embryos | 881 | 7671 | 8552 |
| Larvae | 812 | 7897 | 8709 |
| Pupae | 1069 | 9416 | 10485 |
| Adult females | 932 | 8378 | 9310 |
| Adult males | 1000 | 9347 | 10347 |
| Total | **4694** | **42709** | **47403** |

**(D)** Expression breadth distribution of putatively selected genes in *D. buzzatii*.

| Stages | Selected | Non-selected | Total |
| --- | --- | --- | --- |
| 1 | 106 | 819 | 925 |
| 2 | 166 | 1489 | 1655 |
| 3 | 119 | 1203 | 1322 |
| 4 | 211 | 1407 | 1618 |
| 5 | 611 | 5935 | 6546 |
| Total | **1213** | **10853** | **12066** |

**(E)** Distribution of orphan genes expression in *D. buzzatii* life cycle.

| Stage | Orphans | Non-orphans | Total |
| --- | --- | --- | --- |
| Embryos | 21 | 8531 | 8552 |
| Larvae | 49 | 8660 | 8709 |
| Pupae | 51 | 10434 | 10485 |
| Adult females | 35 | 9275 | 9310 |
| Adult males | 54 | 10293 | 10347 |
| Total | **210** | **47193** | **47403** |

**(F)** Number of orphans and no orphans expressed in one or more stages of *D. buzzatii* life cycle.

| Stages | Orphans | Non-orphans | total |
| --- | --- | --- | --- |
| 1 | 29 | 896 | 925 |
| 2 | 18 | 1637 | 1655 |
| 3 | 11 | 1311 | 1322 |
| 4 | 8 | 1610 | 1618 |
| 5 | 16 | 6530 | 6546 |
| Total | **82** | **11984** | **12066** |

**(G)** Chromosomal location of putatively selected genes detected by site models (SM). The chromosomal location of one of the 772 gene candidates was unknown.

| Chromosome | Selected | Non-selected | Total |
| --- | --- | --- | --- |
| X | 168 | 1259 | 1427 |
| 2 | 154 | 2151 | 2305 |
| 3 | 129 | 1557 | 1686 |
| 4 | 155 | 1653 | 1808 |
| 5 | 161 | 1686 | 1847 |
| 6 | 4 | 25 | 29 |
| Total | **771** | **8331** | **9102** |

**(H)** Chromosomal location of putatively selected genes detected by all models (SM and BSM). The chromosomal locations of two of the 1294 genes were unknown.

| Chromosome | Selected | Non-selected | Total |
| --- | --- | --- | --- |
| X | 260 | 1167 | 1427 |
| 2 | 264 | 2041 | 2305 |
| 3 | 238 | 1448 | 1686 |
| 4 | 245 | 1563 | 1808 |
| 5 | 277 | 1570 | 1847 |
| 6 | 8 | 21 | 29 |
| Total | **1292** | **7810** | **9102** |
